# Cortical Gyrification and Cognitive Decline in the Human Brain With Type 2 Diabetes Mellitus

**DOI:** 10.1002/brb3.70214

**Published:** 2025-01-20

**Authors:** Weiye Lu, Yuna Chen, Zidong Cao, Zhizhong Sun, Wenbin Qiu, Limin Ge, Xin Tan, Yi Liang, Shijun Qiu

**Affiliations:** ^1^ First Clinical Medical College, Guangzhou University of Chinese Medicine Guangzhou China; ^2^ Department of Radiology The First Affiliated Hospital of Guangzhou University of Chinese Medicine Guangzhou China; ^3^ State Key Laboratory of Traditional Chinese Medicine Syndrome Guangzhou China

**Keywords:** cognition, local gyrification index, type 2 diabetes mellitus

## Abstract

**Introduction:**

Type 2 diabetes mellitus (T2DM) is linked to abnormal brain structure and cognitive dysfunction. However, there is a lack of studies conducted to assess the impact of diabetes on cortical gyrification and cognition. The aim of this cross‐sectional study was to assess the potential negative effects of glucose metabolism levels on cognition and cortical gyrification in T2DM.

**Methods:**

The current study comprised 83 patients with T2DM and 60 individuals with normal glucose metabolism (NGM). The calculation of the local gyrification index (LGI) was performed utilizing the FreeSurfer software. Subsequently, between‐group differences were examined through the utilization of analysis of covariance. Multivariable linear regression and mediation models were employed to investigate the relationships among LGI, glucose metabolism and cognition.

**Results:**

Our study found that the mean LGI of the entire brain in individuals with T2DM was lower than that of NGM, and these significant hypogyria were mainly located in the bilateral temporal lobes, including the left superior temporal cortex, left transverse temporal cortex, and bilateral temporal pole, with the greatest effect size in the left temporal pole (*p* = 5.7×10^−7^, Cohen's *f* ^2^ = 0.169). In addition, the relationship between fasting blood glucose and working memory was mediated by the LGI in the right temporal pole.

**Conclusion:**

Our experiment suggests t**hat the de**creased LGI in the right temporal pole explains poorer working memory performance in patients with T2DM.

## Graphical abstract

The relationship between fasting blood glucose and working memory in patients with type 2 diabetes is partially mediated by cortical gyrification of the right temporal pole. This study suggests that strengthening blood glucose level management through lifestyle intervention may be an effective method to alleviate cognitive decline in elderly patients with type 2 diabetes.

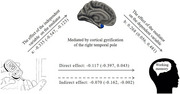

## 1 Introduction

According to the International Diabetes Federation, 537 million individuals aged 20–79 years are now living with diabetes, representing 10.5% of the world's population in this age range (Magliano and Boyko 2021).About 90%–95% of all cases of diabetes are type 2 diabetes mellitus (T2DM), often known as “adult‐onset diabetes” (American Diabetes Association 2015). is currently a substantial body of research that connects type 2 diabetes to an elevated risk of structural brain abnormalities on MRI, including greater levels of white matter hyperintensities and lacunar infarcts and lower cerebral gray and white matter volume (van Sloten et al. 2020; van Harten et al. 2006; van Agtmaal et al. 2018), which further contributes to the decline in human cognitive function (van Gennip et al. 2021; Mankovsky et al. 2018). The pathogenesis of type 2 diabetes–related cognitive decline is integrative, multifactorial, and not yet fully understood. Research has found that type of diabetes, age of onset, progression, blood glucose, obesity, hypertension, and microvascular or/and macrovascular complications may be connected to the development of cognitive impairment (van Duinkerken and Ryan 2020). Given the aging and growth of the T2DM population, there is an imperative need to investigate the potential mechanisms that contribute to cognitive impairment patients in this patient population.

Furthermore, apart from microvascular or macrovascular complications associated with diabetes, T2DM has also been linked to morphological alterations in brain structure. According to the results of the voxel‐based morphometry study, individuals with T2DM exhibited indicators of brain atrophy. This included a decline in total and regional gray matter and white matter volumes (Moheet, Mangia, and Seaquist 2015; Moran et al. 2013). In comparison to healthy controls, patients with T2DM consistently exhibited decreased total cortical surface area, total cortical volume, and mean cortical thickness, but this effect was only observed in the right hemisphere (Brundel et al. 2010; Peng et al. 2015). Similar to this hypothesis, the Alzheimer's Disease Neuroimaging Initiative (ADNI) recently reported that patients with T2DM were associated with lower bilateral frontal and parietal cortical thickness in an elderly study population (Moran et al. 2015). It is worth noting that in case of cortical gyrification as a superficial feature, only a few studies have been evaluated in patients with T2DM, and the conclusions are inconsistent.

Cortical gyrification is recognized as early neurodevelopmental imaging biomarkers that are primarily determined prenatally (Armstrong et al. 1995). There is a proposition that during the prenatal stage, the development of the formation of cortical connectivity leads to the emergence of fiber tension. This tension causes highly linked regions to be drawn together, resulting in the formation of bulging gyri. Conversely, regions with sparser connections tend to drift apart and become separated by inward sulci (White and Hilgetag 2011). The local gyrification index (LGI) is an indicator of the degree of folding, which is the ratio between the outer surface of the total cortex and the exposed part of the outer surface (Zilles, Palomero‐Gallagher, and Amunts 2013). Gyrification abnormalities, such as polymicrogyria and pachygyria, have the potential to induce modifications in brain functionality, resulting in observable deficits in language and cognitive abilities. A large‐sized prospective cohort study comprising 4397 middle‐aged and elderly participants reported that lower age and higher cognitive scores were associated with higher global gyrification (Lamballais et al. 2020). Similarly, in Alzheimer's patients, greater insula cortex folding was found to directly correspond with improved memory and semantic fluency (Núñez et al. 2020). However, given that cortical gyrification is linked with cognitive performance, it is unknown whether the accelerated cognitive decline of T2DM patients is correlated with a pattern of altered cortical gyrification. Thus far, only two studies have evaluated the pattern of cortical gyrification alterations in T2DM, and the results are conflicting (Crisóstomo et al. 2021; Shao et al. 2022).

In the present study, we sought to determine whether T2DM is correlated with LGI. The relationship between LGI and clinical features and neurocognitive measures was then further explored. We hypothesized that T2DM subjects would have a decrease in mean LGI compared to the healthy controls and would exhibit this change in specific brain regions. Additionally, it was postulated that the aforementioned brain regions would exhibit a correlation with a decrease in cognitive performance within distinct domains.

## 2 Materials and Methods

### 2.1 Participants

The present study received approval from the Ethics Committee of the First Affiliated Hospital of Guangzhou University of Chinese Medicine, Guangzhou, China (No. k[2023]146). All participants were between the ages of 35 and 70, and those with type 1 diabetes or other types of diabetes were excluded from the study. All individuals included in the study were right‐handed and had written informed consent. T2DM status was defined according to the American Diabetes Association 2021 diagnostic criteria (American Diabetes Association 2021). Individuals with T2DM were recruited from the endocrine inpatient and outpatient facilities of the First Hospital of Guangzhou University of Traditional Chinese Medicine. Participants with normal glucose metabolism (NGM) were recruited from the community through poster advertisement at the same time.

In addition, participants in this study will be excluded if they meet one of the following exclusion criteria: (1) left‐handed; (2) unstable glucose control; (3) substance abuse; (4) history of brain tumor, trauma, surgery, or other organic disease; (5) met the diagnostic criteria for depression, schizophrenia, schizoaffective disorder, obsessive‐compulsive disorder, or anxiety disorder; and (6) pregnancy, breastfeeding, or other contraindications to MRI during the study period.

In total, 150 participants completed the baseline survey between November 2020 and May 2023. Seven MRI scans were excluded due to pathology (*n* = 1), metal artifacts (*n* = 1), or inadequate scan quality (*n* = 5), resulting in 143 MRI scans included in the final analysis (including 83 T2DM patients and 60 normal glucose metabolism).

### 2.2 Clinical and Cognitive Assessments

Data on sociodemographic and clinical characteristics were gathered for all study participants. Demographics included age, gender, education, and disease duration. Biochemical information included FBG, HbA1c, body mass index (BMI), systolic and diastolic blood pressure, homeostatic model assessment of insulin resistance (HOMA‐IR), fasting insulin, and ratio of total cholesterol to high‐density lipoprotein.

### 2.3 Cognition

General cognitive ability was assessed using the Mini‐Mental State Examination (MMSE) and the Montreal Cognitive Assessment (MoCA). Several cognitive subdomains, including episodic memory, working memory, executive function, information processing speed, and attention were assessed by the following tests: Auditory Verbal Learning Test, the Digital Span Test (backward), Trail Making Test (including parts A and B), and Digit Symbol Substitution Test. Thorough explanations of tests and cognitive domain assessments can be found in Appendix E1.

### 2.4 Brain MRI

MRI data were collected using a 3.0T Siemens MAGNETOM Prisma clinical MRI scanner with a 64‐channel head coil. Earplugs and cushions are employed to minimize aural impact and head movement, respectively. The MRI protocol consisted of structural scans used for radiological assessment, which included T1‐weighted, T2‐weighted, and fluid‐attenuated inversion recovery sequences. T1 images were obtained using magnetization‐prepared rapid gradient echo sequences: TR, 2530 ms; TE, 2.98 ms; slices, 192; thickness, 1 mm; FOV, 224 mm × 256 mm; flip angle, 7 ^°^; and voxel size, 1 mm × 1 mm × 1 mm.

### 2.5 Imaging Processing

**FIGURE 1:**
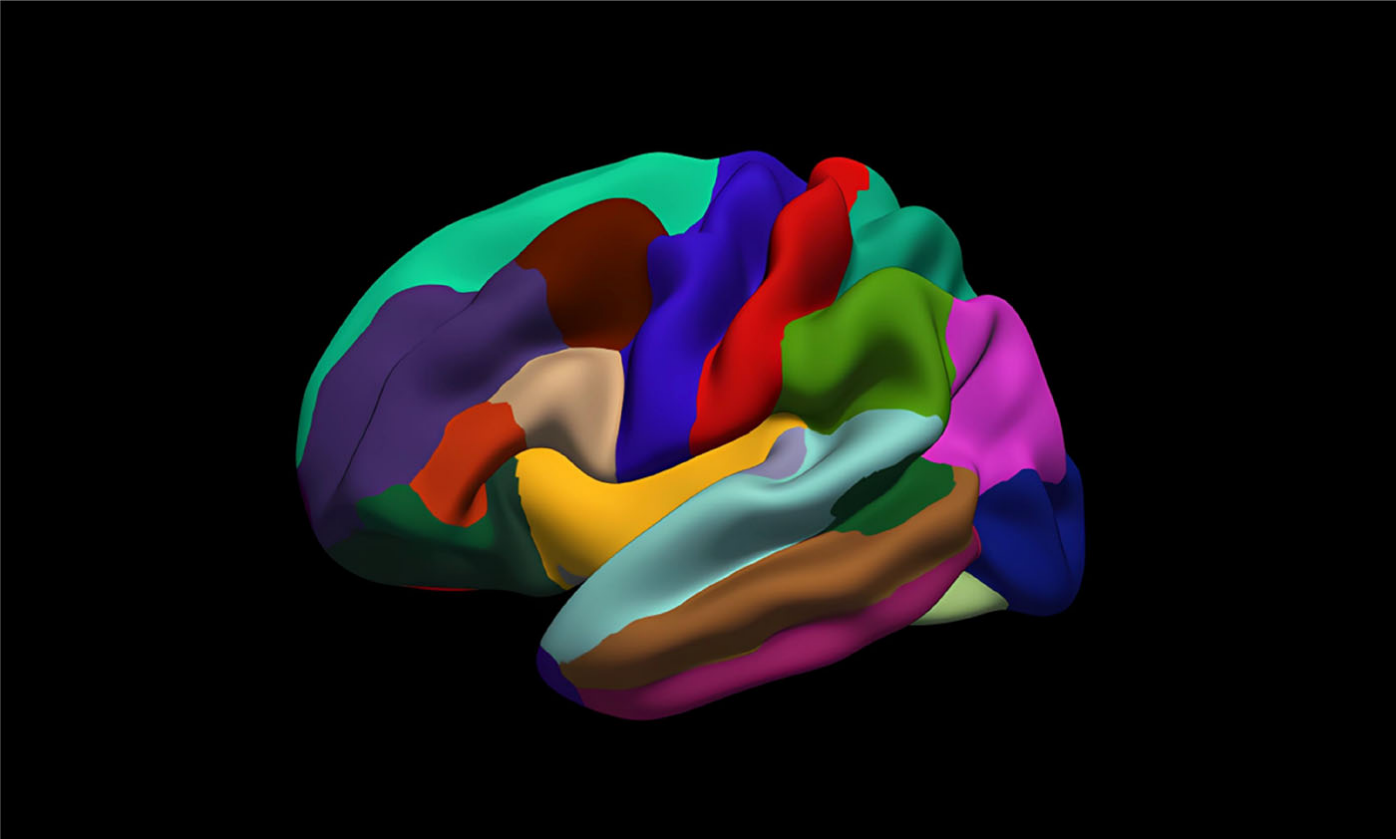
The Desikan–Killiany atlas divides each hemisphere into 33 cortical regions.

The FreeSurfer software package was used to preprocess the 3D T1 pictures using conventional and automatic reconstruction approaches (version 6.0, http://surfer.nmr.harvard.edu). The processing flow involves several steps, including the removal of nonbrain tissue, automatic transformation to Talairach space, normalization of intensity, and segmentation of gray matter/white matter tissue to generate a white mesh and a pial mesh. Each hemisphere of the mesh has about 150,000 vertices. We determined LGI values using a previously established standard formula (Fischl and Dale 2000). Using an automated FreeSurfer procedure, the calculation of the LGI was performed using a series of consecutive cortical reconstruction procedures. This involved determining the ratio between the surface area of sulcal and buried regions and the surface area of the outer hull, which serves as an indicator of the level of cortical folding inside specific three‐dimensional regions of interest on a spherical surface (Nanda et al. 2014). The Desikan–Killiany atlas (Desikan et al. 2006) was utilized to partition each hemisphere into 33 cortical regions, as shown in Figure 1 and Table 2. The average LGI value across the subregions that make up the Desikan–Killiany atlas was used to derive the mean LGI value for the entire brain. In the analyses, 66 cortical regions' LGI values from both hemispheres were calculated.

### 2.6 Statistical Analyses

Independent *t* tests (for normally distributed continuous variables) or *χ* ^2^ tests (for categorical variables) were utilized to determine the distribution of the study population's general characteristics, which were expressed as mean ± SD, percentage, or median (interquartile spacing). *p* < 0.05 was considered statistically different. A one‐way analysis of covariance (ANCOVA) was employed to compare the LGI values of T2DM and NGM groups. In this analysis, the groups were the independent variables, the dependent variables were 66 LGIs extracted from each cortical region, and age, gender, education level, systolic blood pressure, and ratio of total cholesterol to high‐density lipoprotein were taken as covariates. We used the Bonferroni correction for multiple comparisons in all analyses (i.e., *p* < 0.05/66 = 0.000758).

#### 2.6.1 Multivariable Linear Regression

We examined the correlation between glucose metabolism, LGI, and cognitive function using multivariate linear regression, controlling for age, gender, education level, systolic blood pressure, and ratio of total cholesterol to high‐density lipoprotein. The Statistical Package for Social Sciences (IBM, SPSS, version 27) was used for all statistical analysis.

#### 2.6.2 Mediation Analysis

With the understanding that LGI has an impact on cognition, simple mediation models were utilized to investigate if specific LGI alterations are on the probable causation pathway of the link between glucose metabolism and cognition. To calculate bias‐corrected 95% CIs, we used bootstrapping (5000 samples) and the PROCESS statistical package for SPSS (Hayes 2013). The statistical analyses were adjusted for age, gender, level of education, systolic blood pressure, and ratio of total cholesterol to high‐density lipoprotein.

## 3 Results

### 3.1 General Characteristics and Cognitive Assessment

The characteristics of the sample population in NGM and T2DM are displayed in Table 1. Age, gender, BMI, and alcohol and smoking habits were matched between T2DM and NGM groups. Patients with T2DM were more likely to have higher education years, systolic blood pressure, total cholesterol to HDL ratio, FBG, HOMA‐IR, and HbA1c compared to NGM (all *p* < 0.05). Among the 83 patients with T2DM, 15 (20.5%) used insulin and 22 (26.5%) took antihypertensive drugs. The average duration of disease was 5.8 ± 5.3 years.

**TABLE 1:** Participants’ characteristics and cognitive test scores.

	T2DM (*n *= 83)	NGM (*n* = 60)	*p* value (*t*, *z*, *χ^2^*)
Demographics			
Age	52.6 ± 6.1	51.3 ± 5.6	0.203 (*t *= 1.280)
Sex, male (%)	44 (53.0)	27 (46.5)	0.450 (*χ* ^2 ^= 0.450)
Education (years)	11 (8, 14)	9 (7.8, 9)	< 0.001 (*z *= 3.380)*
Cardiovascular risk factors			
BMI (kg/m^2^)	23.9 ± 3.4	23.6 ± 4.1	0.597 (*t *= 0.529)
Systolic blood pressure (mmHg)	130.1 ± 16.4	123.8 ± 13.6	0.017 (*t *= 2.422)*
Diastolic blood pressure (mmHg)	84.1 ± 11.1	82.0 ± 8.8	0.225 (*t *= 1.218)
Ratio of total cholesterol to HDL	4.4 ± 1.3	3.7 ± 1.0	0.002 (*t* = 3.210)*
Glucose metabolism			
FBG (mmol/L)	9.7 ± 2.9	5.1 ± 0.4	< 0.001 (*t = *13.917)*
HbA1c (%)	8.9 ± 2.4	5.1 ± 0.4	< 0.001 (*t = *13.758)*
Diabetes duration (years)	5.8 ± 5.3	—	—
Fasting insulin (mIU/mL)	11.1 ± 9.2	9.8 ± 5.6	0.368 (*t* = 0.903)
HOMA‐IR	4.64 ± 4.4	2.2 ± 1.4	< 0.001 (*t = *4.591)*
Medication use (%)			
Oral hypoglycemic medication	58 (69.9)	—	—
Insulin and oral hypoglycemic medication	17 (20.5)	—	—
None	8 (9.6)	—	—
Antihypertension medication	12 (27.7)	—	—
Lifestyle factors (%)			
Smoking habits, yes (%)	14 (16.9)	11 (18.9)	0.749 (*χ^2 = ^* 0.103)
Alcohol consumption, yes (%)	10 (12.0)	6 (10.3)	0.753 (*χ^2 = ^* 0.099)
Cognitive score			
MoCA < −1.5 SD (%)	10 (12.0)	1 (1.7)	0.027 (*χ^2^* ^ = ^0.450)
MoCA	24.7 ± 3.2	26.2 ± 2.7	0.003 (*t* = −3.079)*
MMSE	27.5 ± 1.9	28.4 ± 1.6	0.003 (*t = *−2.998)*
Episodic memory	−0.12 ± 1.1	0.18 ± 0.7	0.058 (*z* = −1.914)
Working memory	−0.19 ± 1.0	0.27 ± 0.9	0.006 (*z* = −2.791)*
Executive function and attention	−0.11 ± 1.0	0.15 ± 0.9	0.133 (*z = *−1.510)
Information processing speed	−0.12 ± 1.1	0.17 ± 0.8	0.095 (*z* = −1.679)

*Note*: Data were displayed as means ± SD or numbers (proportions) or median (interquartile range).

Abbreviations: BMI, body mass index; FBG, fasting blood glucose; HbA1c, hemoglobin A1c; HDL, high‐density lipoprotein; HOMA‐IR, homeostatic model assessment of insulin resistance; MMSE, Mini‐Mental State Examination; MoCA, Montreal Cognitive Assessment; NGM, normal glucose metabolism; T2DM, type 2 diabetes mellitus.

*p < 0.05 was considered significant.

The T2DM group exhibited significantly lower general cognition scores on the MoCA and MMSE compared to the NGM group (all *p *< 0.05). Except for working memory, there were no statistical differences between the two groups in other cognitive subdomains.

### 3.2 Between‐Group Differences in LGI

**FIGURE 2:**
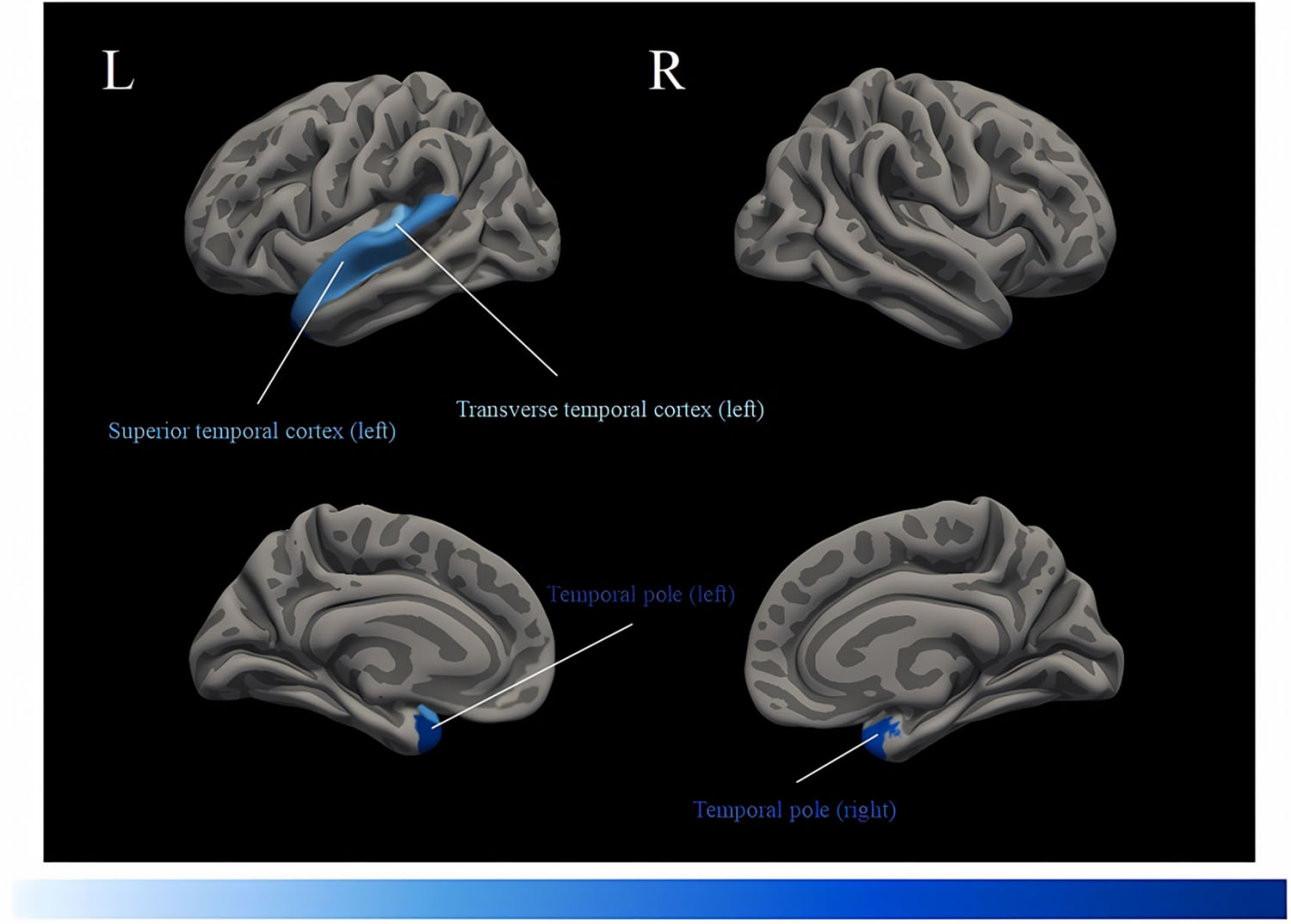
Cortical regions with significantly decreased gyrification in type 2 diabetes, including left transverse temporal cortex, left superior temporal cortex, and bilateral temporal poles (all *p* < 0.05/66). The darker color on the blue bar indicates a higher Cohen's *f* ^2^ value for the reduction of gyrification.

**FIGURE 3:**
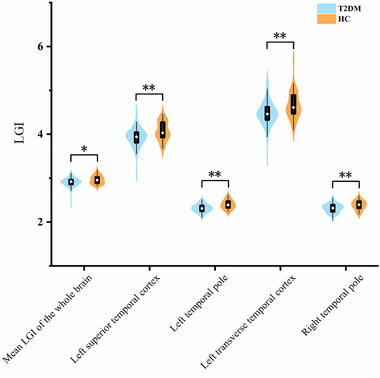
Mean local gyrification index (LGI) adjusted for age, sex, education level, systolic blood pressure, and the ratio of total cholesterol to high‐density lipoprotein and was displayed as mean with one standard deviation. * denotes *p* < 0.05 and ** denotes LGI with significant between‐groups differences after the Bonferroni correction (*p* < 0.000758).

Table 2 summarizes the findings of the whole‐brain LGI values. On a macroscopic level, it was shown that the average LGI of the entire brain was notably reduced in individuals with T2DM in comparison to NGM (T2DM = 2.91 ± 0.10; NGM = 2.96 ± 0.09; *F*_(1, 137)_ = 7.940; *p* = 0.006; Cohen's *f* ^2^ = 0.056). Patients with T2DM revealed significantly lower LGIs than NGM in four cortical regions out of 66 in the bilateral hemispheres (Figures 2 and 3), including the left superior temporal cortex (T2DM = 3.93 ± 0.21; NGM = 4.07 ± 0.21; *F*_(1, 137)_ = 14.830; *p* = 0.00018; Cohen's *f* ^2^ = 0.100), left temporal pole (T2DM = 2.31 ± 0.09; NGM = 2.40 ± 0.10; *F*_(1, 137)_ = 29.119; *p* = 3.0 × 10^−7^; Cohen's *f* ^2^ = 0.169), left transverse temporal cortex (T2DM = 4.47 ± 0.28; NGM = 4.65 ± 0.29; *F*_(1, 137) _= 14.038; *p* = 0.00027; Cohen's *f* ^2^ = 0.095), and right temporal pole (T2DM = 2.31 ± 0.10; NGM = 2.38 ± 0.10; *F*_(1, 137)_ = 21.371; *p* = 9.0 × 10^−6^; Cohen's *f* ^2^ = 0.138). The statistical significance of these findings remained significant after the Bonferroni correction (all *p* < 0.000758). There was no observed significant increased LGI in any cortical region among T2DM compared to NGM.

**TABLE 2:** Comparison of the local gyrification index between type 2 diabetes and normal glucose metabolism.

	T2DM ( *n = * 83)		NGM ( *n* = 60)		T2DM vs. NGM		
Cortical regions	Mean	SD	Mean	SD	*F*_(1,137)_	*p* value	Cohen's *f* ^2^
Left hemisphere							
Caudal anterior cingulate cortex	1.85	0.09	1.90	0.10	3.915	0.050	0.028
Caudal middle frontal gyrus	3.06	0.16	3.09	0.15	0.996	0.320	0.007
Cuneus	2.91	0.19	2.93	0.16	0.050	0.824	3.7 × 10**^−^** ^4^
Entorhinal cortex	2.54	0.12	2.56	0.13	2.183	0.142	0.016
Fusiform gyrus	2.64	0.11	2.67	0.10	1.286	0.259	0.010
Inferior parietal cortex	3.19	0.13	3.24	0.12	1.928	0.167	0.014
Inferior temporal gyrus	2.65	0.12	2.69	0.12	1.584	0.210	0.012
Isthmus of cingulate cortex	2.68	0.17	2.74	0.18	3.414	0.067	0.025
Lateral occipital cortex	2.56	0.10	2.59	0.11	0.875	0.351	0.006
Lateral orbitofrontal cortex	2.53	0.10	2.58	0.11	9.645	0.002	0.067
Lingual gyrus	2.76	0.14	2.79	0.14	0.475	0.492	0.004
Medial orbitofrontal cortex	2.07	0.09	2.12	0.09	4.178	0.043	0.030
Middle temporal gyrus	3.25	0.18	3.33	0.18	4.014	0.047	0.029
Parahippocampal gyrus	2.82	0.14	2.82	0.15	0.006	0.941	4.2 × 10**^−^** ^5^
Paracentral lobule	2.29	0.11	2.35	0.11	4.626	0.033	0.033
Pars opercularis	4.02	0.28	4.10	0.26	6.442	0.012	0.046
Pars orbitalis	2.89	0.17	2.87	0.13	0.359	0.550	0.003
Pars triangularis	3.60	0.23	3.65	0.25	3.300	0.072	0.024
Pericalcarine cortex	2.79	0.17	2.81	0.15	0.010	0.919	7.7 × 10**^−^** ^5^
Postcentral gyrus	3.42	0.16	3.48	0.13	4.173	0.043	0.030
Posterior cingulate cortex	2.14	0.13	2.18	0.14	1.903	0.170	0.014
Precentral gyrus	3.33	0.15	3.41	0.13	6.631	0.011	0.047
Precuneus	2.85	0.17	2.90	0.16	1.796	0.183	0.013
Rostral anterior cingulate cortex	1.99	0.08	2.05	0.09	7.938	0.006	0.056
Rostral middle frontal gyrus	2.70	0.13	2.71	0.10	0.529	0.468	0.004
Superior frontal gyrus	2.14	0.07	2.17	0.08	1.924	0.168	0.014
Superior parietal cortex	2.94	0.13	2.98	0.13	1.570	0.212	0.012
**Superior temporal cortex**	3.93	0.21	4.07	0.21	**14.830**	**1.8 × 10** ^−4^	0.100
Supramarginal gyrus	3.50	0.15	3.56	0.14	2.807	0.096	0.021
Frontal pole	2.04	0.09	2.07	0.09	1.812	0.181	0.013
**Temporal pole**	2.31	0.09	2.40	0.10	**29.119**	**3.0 × 10** ^−^ **^7^**	0.179
**Transverse temporal cortex**	4.47	0.28	4.65	0.29	**14.038**	**2.7 × 10** ^−^ **^4^**	0.095
Insula	4.07	0.24	4.20	0.27	10.674	0.001	0.074
Right hemisphere							
Caudal anterior cingulate cortex	1.92	0.10	1.94	0.11	0.934	0.336	0.007
Caudal middle frontal gyrus	3.06	0.15	3.10	0.12	0.554	0.458	0.004
Cuneus	3.07	0.19	3.12	0.20	1.504	0.222	0.011
Entorhinal cortex	2.58	0.12	2.61	0.13	2.703	0.103	0.020
Fusiform gyrus	2.63	0.11	2.64	0.11	0.131	0.718	0.001
Inferior parietal cortex	3.17	0.13	3.22	0.19	1.920	0.168	0.014
Inferior temporal gyrus	2.59	0.10	2.61	0.08	0.585	0.446	0.004
Isthmus of cingulate cortex	2.78	0.17	2.84	0.20	2.268	0.134	0.017
Lateral occipital cortex	2.57	0.10	2.59	0.12	0.740	0.391	0.005
Lateral orbitofrontal cortex	2.52	0.09	2.56	0.09	5.110	0.025	0.037
Lingual gyrus	2.84	0.14	2.89	0.15	4.286	0.040	0.031
Medial orbitofrontal cortex	2.11	0.08	2.14	0.09	1.686	0.196	0.012
Middle temporal gyrus	3.20	0.17	3.25	0.17	2.196	0.141	0.016
Parahippocampal gyrus	2.86	0.15	2.86	0.17	0.023	0.880	1.7 × 10^−4^
Paracentral lobule	2.30	0.10	2.37	0.11	6.911	0.010	0.049
Pars opercularis	4.04	0.25	4.15	0.28	7.094	0.009	0.050
Pars orbitalis	2.91	0.17	2.92	0.16	0.251	0.618	0.002
Pars triangularis	3.65	0.26	3.69	0.27	2.042	0.155	0.015
Pericalcarine cortex	2.93	0.17	2.97	0.18	2.636	0.107	0.019
Postcentral gyrus	3.37	0.16	3.44	0.14	3.966	0.048	0.029
Posterior cingulate cortex	2.15	0.10	2.20	0.14	2.069	0.153	0.015
Precentral gyrus	3.31	0.15	3.38	0.12	4.664	0.033	0.034
Precuneus	2.99	0.18	3.06	0.19	3.840	0.052	0.028
Rostral anterior cingulate cortex	2.06	0.09	2.11	0.10	2.967	0.087	0.022
Rostral middle frontal gyrus	2.69	0.12	2.74	0.11	5.386	0.022	0.039
Superior frontal gyrus	2.21	0.08	2.25	0.07	2.027	0.157	0.015
Superior parietal cortex	2.92	0.12	2.98	0.12	6.444	0.012	0.046
Superior temporal cortex	3.95	0.22	4.05	0.24	6.872	0.010	0.049
Supramarginal gyrus	3.45	0.15	3.51	0.13	2.080	0.152	0.015
Frontal pole	2.11	0.10	2.13	0.09	0.217	0.642	0.002
**Temporal pole**	2.31	0.10	2.38	0.10	**21.371**	**9.0 × 10** ^−^ **^6^**	0.138
Transverse temporal cortex	4.49	0.27	4.66	0.30	9.597	0.002	0.067
Insula	4.08	0.25	4.18	0.28	3.868	0.051	0.028
Mean LGI of the whole brain	2.91	0.10	2.96	0.09	7.940	0.006	0.056

*Note*: Local gyrification index (LGI) is displayed as mean with one standard deviation. Significant between‐groups differences after the Bonferroni correction (*p* < 0.05/66 = 0.000758) are shown in bold.

Abbreviations: NGM, normal glucose metabolism; SD, standard deviation; T2DM, type 2 diabetes mellitus.

### 3.3 Relationship Between Differential LGI and Clinical Characteristics Among T2DM Patients

#### 3.3.1 Association Between Glucose Metabolism and Cognitive Function

Higher FBG levels were correlated with lower MoCA scores (st*β* = −0.332 [95% CI:−0.516, −0.147]; *p* < 0.001), poorer episodic memory scores (st*β* = −0.255 [95% CI: −0.450, −0.059]; *p* = 0.011), poorer working memory scores (st*β* = −0.246 [95% CI: −0.457, −0.036]; *p* = 0.022), and poorer information processing speed scores (st*β* = −0.192 [95% CI: −0.376, −0.008]; *p* = 0.041). Besides, higher levels of HbA1c were associated with lower MoCA scores (st*β* = −0.343 [95% CI: −0.525, −0.161]; *p* < 0.001), lower episodic memory scores (st*β* = −0.245 [95% CI: −0.439, −0.050]; *p* = 0.014), and lower information processing speed scores (st*β* = −0.257 [95% CI: −0.435, −0.079]; *p* = 0.005; Table 3).

**TABLE 3:** Multivariable‐adjusted associations of glucose metabolism with cognitive function.

	Fasting blood glucose		HbA1c		Fasting insulin	
Cognition test	St*β* [95%CI]	*p*	St*β* [95%CI]	*p*	St*β* [95%CI]	*p*
MoCA	**−0.332 (−0.516, −0.147)**	**< 0.001**	**−0.343 (−0.525, −0.161)**	**< 0.001**	0.110 (−0.077, 0.298)	0.245
Episodic memory	**−0.255 (−0.450, −0.059)**	**0.011**	**−0.245 (−0.439, −0.050)**	**0.014**	−0.017 (−0.210, 0.176)	0.860
Working memory	**−0.246 (−0.457, −0.036)**	**0.022**	−0.080 (−0.295, 0.135)	0.461	−0.082 (−0.288, 0.123)	0.427
Executive function and attention	0.114 (−0.106, 0.334)	0.307	0.189 (−0.027, 0.405)	0.085	−0.153 (−0.360, 0.054)	0.145
Information processing speed	**−0.192 (−0.376, −0.008)**	**0.041**	**−0.257 (−0.435, −0.079)**	**0.005**	0.079 (−0.099, 0.257)	0.379

*Note*: Data are displayed as standardized *β* coefficients and 95% CI. Significant group differences are presented in bold.

Abbreviation: MoCA, Montreal Cognitive Assessment.

#### 3.3.2 Association Between Glucose Metabolism and Differential LGI

Among the four LGI with statistical differences, higher FBG levels were associated with smaller LGI in the right temporal pole (st*β* = −0.335 [95% CI: −0.547, −0.123]; *p* = 0.002; Table E1).

#### 3.3.3 Association Between Differential LGI and Cognitive Function

Among the four LGIs with statistical differences, higher LGI in the right temporal pole was associated with higher working memory scores (st*β* = 0.268 [95% CI: 0.056, 0.481]; *p* = 0.014) and higher information processing speed scores (st*β* = 0.243 [95% CI: 0.060, 0.427]; *p* = 0.010; Table E2).

### 3.4 Mediation Analysis

**FIGURE 4:**
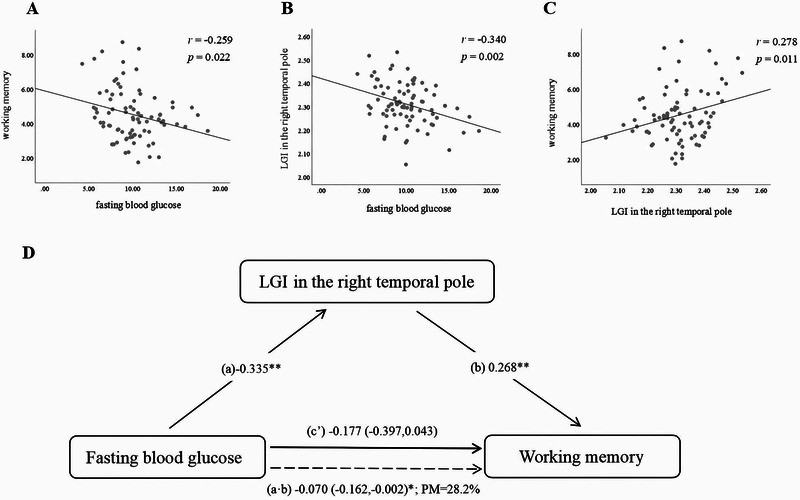
(A) Fasting blood glucose was negatively associated with working memory (*r* = −0.259; *p* = 0.022). (B) Fasting blood glucose was negatively correlated with local gyrification index (LGI) in the right temporal pole (*r* = −0.340; *p* = 0.002). (C) LGI in the right temporal pole was positively correlated with working memory (*r* = 0.278; *p* = 0.011). (D) Diagram illustrates associations between fasting blood glucose, LGI in right temporal pole, and working memory and mediation by LGI in right temporal pole. Solid lines indicate direct effects (c’); dashed lines indicate indirect effects (a·b) and percentage mediated (percentage mediated [PM] = indirect/total). All correlations were adjusted for age, gender, years of education, systolic blood pressure, and ratio of total cholesterol to high‐density lipoprotein. * = *p* < 0.05 and ** = *p *< 0.01.

LGI in the right temporal pole mediated for 28.2% (indirect effect: st*β* = −0.070 [95% CI: −0.162, −0.002], *p* < 0.05) the association between FBG levels and working memory scores (Figure 4 and Table E3).

### 3.5 Additional Analyses

It is well‐known that hypoglycemic drugs can have an effect on the brain. When we additionally adjusted for different hypoglycemic treatments, only bilateral temporal poles in T2DM patients showed hypogyria compared with NGM patients (Table E4). Furthermore, analyzing the relationship between age and duration of type 2 diabetes and population‐based LGI via multiple linear regression. We found that the mean LGI was negatively correlated with age (*r* = −0.311; *p* < 0.001). However, we did not observe a significant correlation between mean LGI and duration in T2DM.

## 4 Discussion

The current study revealed that the average LGI of the entire brain in T2DM was found to be lower compared to that of NGM. Notably, these statistically significant reductions in gyrification were primarily localized in the bilateral temporal lobes, specifically in regions such as the left transverse temporal cortex, left superior temporal cortex, and bilateral temporal pole. Among these areas, the left temporal pole exhibited the greatest effect size. Regarding interrelations between glucose metabolic status, LGI within the T2DM group, and cognitive function, we found that higher levels of short‐term marker glucose, FBG, were correlated with smaller LGIs in the right temporal pole, as well as poorer performance of general cognition, episodic memory, working memory, and information processing speed. Furthermore, we found that greater levels of HbA1c, a long‐term biomarker of glycemic management, were also correlated with a decline in general cognition, episodic memory, and information processing speed. The relationship between FBG and working memory scores was partly mediated by the LGI in the right temporal pole.

In the present study, the T2DM group showed significant hypogyria in the temporal lobes, and no cortical regions exhibited greater LGIs compared to the NGM group. In partial conflict with our findings, Crisóstomo et al. (2021) found clusters of elevated cortical gyrification index in the posterior lobe, temporal lobes, occipital lobe, and parietal lobe and decreased cortical gyrification index in the temporal lobe. This may be due to the different ways of quantifying the characteristics of gyrification, since Crisóstomo et al.’s (2021) study calculated gyrification through curvature‐based measurements (Luders et al. 2006). But it is uncertain whether the degree of curvature and a biologically meaningful correlation, such as cortical volume or surface, are directly associated, making it difficult to interpret the curvature (Schaer et al. 2012). The proportion of the buried cortical surface area to the outer convex surface area for each parcellated cortical region, expressed as the buried cortical surface area (mm^2^) divided by the outer convex surface area (mm^2^) (Choi et al. 2022), was used in the current study to calculate the mean LGI. Gyrification index in spherical three‐dimensional zones of interest was iteratively quantified to determine the measurement (Schaer et al. 2008), which may increase sensitivity to detect gyrification abnormalities. However, there was also a common pattern of LGI alteration, that is, compared to the NGM group, the T2DM groups shared the same significant hypogyria in the right temporal pole (Crisóstomo et al. 2021). Another T2DM‐related study reported decreased gyrification in the bilateral insula (Shao et al. 2022), which differs from our findings and suggests that additional cortical regions may also be impacted by T2DM. Furthermore, the decreased LGIs in left superior temporal cortex and transverse temporal cortex are unilateral findings. We speculate that this phenomenon is due to lateralization of brain function as the participants were right‐handed (Güntürkün, Ströckens, and Ocklenburg 2020).

Decreased gyrification in T2DM patients may be explained by the widely accepted tension‐based morphogenetic hypothesis. The connectivity of the cerebral cortex is closely correlated with the process of gyrification. That is to say, tension along axons in the white matter (including connections between cortex and various subcortical nuclei, as well as specific cortico‐cortical projections) provides the main driving force for cortical folding (Zilles, Palomero‐Gallagher, and Amunts 2013; van Essen 1997). Previous studies have reported that white matter microstructural integrity is disrupted in patients with prediabetes and T2DM (Jing et al. 2022; Hsu et al. 2012; Zhang et al. 2014; Vergoossen et al. 2020). On the other hand, the differential tangential growth theory suggests that numerous characteristics of cortical folding can be explained by considering cortical thickness and the growth rate of the cortex in relation to the subcortical layers (Richman et al. 1975; Garcia, Kroenke, and Bayly 2018). To date, previous structural reports have described alterations in the temporal lobe of individuals with T2DM, revealing a reduction in both volume and cortical thickness in specific regions, including the temporal pole, superior temporal gyrus, and transverse temporal gyrus (Yao et al. 2021). More importantly, those who have T2DM exhibit a unique pattern of structural covariation, that is, a sequence of gray matter volume alterations from the right temporal pole to regions of the limbic–cerebellar–striatal–cortical network (Zhang et al. 2022). Additionally, we discovered that the average LGI decreases with age, which is consistent with prior findings (Madan 2021). Based on these theories, the decreased LGI in T2DM patients may be linked to the alteration of brain morphology and white matter integrity caused by long‐term exposure to hyperglycemia. However, in the present study, we did not find a relationship between the average LGI and disease duration of T2DM. We speculate that this may be related to inaccurate self‐reporting of disease course by patients with T2DM, as the onset of T2DM may begin before the patients realize it.

Compared to the NGM group, patients with T2DM demonstrated a significant decline in general cognition and working memory, and a downward trend in both episodic memory and information processing speed was also observed. There was a negative correlation seen between the cognitive performance of individuals with T2DM and their fasting blood glucose and HbA1c levels. There has been a lot of research and clinical attention on cognitive impairment in T2DM patients (Geijselaers et al. 2015). Individuals with T2DM exhibit a 1.5‐fold elevated susceptibility to developing dementia in comparison to those without the disease, as well as an accelerated rate of cognitive decline and subtle cognitive impairment (Biessels et al. 2014). Consistent with our findings, a meta‐analysis including 15 articles reported altered cognitive performance in individuals with T2DM, showing decrements in memory function, speed of processing, and executive function (Sadanand, Balachandar, and Bharath 2016). Similar to this, Moheet, Mangia, and Seaquist (2015) reported that T2DM was linked to mild‐to‐moderate cognitive dysfunction, mainly in memory function. Further exploring, we also demonstrated that more severe impaired fasting blood glucose and long‐term higher glucose levels were correlated with decreased cognitive function, which was in line with a previous study (Kerti et al. 2013). Finally, we confirmed that the decreased LGI in the right temporal pole plays a mediating role in related working memory impairment in individuals with T2DM through mediating analysis.

Given the lack of clarity surrounding the specific mechanisms of cognitive impairment in T2DM, it is important to identify a brain morphological marker that may provide a potential link. Kerti et al. (2013) reported that hippocampal volume and microstructure mediated the relationship between HbA1c and glucose levels and memory consolidation scores. Our study's mediation analysis suggests that LGI alteration in the right temporal pole mediates the link between fasting glucose levels and working memory in T2DM. In this study, we discovered that the LGI of the right temporal pole is most closely related to diabetes‐related cognitive impairment rather than other cerebral cortex. Previous structural reports have also mentioned the volume changes of the right temporal pole in T2DM patients, showing a sequence of GMV changes from the right temporal pole to the limbic–cerebellar–striatal–cortical network with the prolongation of the course of disease (Zhang et al. 2022). Wennberg et al. (2016) also found that the increase of FBG was statistically significant correlated with the decrease of the thickness of temporal pole and parahippocampal gyrus. Some early neurodegenerative diseases, such as amnesia, mild cognitive impairment, and Alzheimer's disease, are correlated with the temporal pole (Herlin, Navarro, and Dupont 2021), which may explain memory impairment in diabetes diseases. According to the theory of differential tangential growth, we speculate that the decline in LGI in the right temporal pole is due to a unique pattern of morphological changes in their brains, that is, a single or joint alteration in both gray matter volume and cortical thickness, which ultimately leads to a decline in working memory in T2DM patients.

When interpreting our findings, a few limitations need to be kept in mind. Since this was a cross‐sectional investigation, it is unable to verify whether hypogyria in specific cortical regions leads to accelerated cognitive decline in T2DM. To completely comprehend the developmental alterations of LGI in patients with T2DM, it is advised that prediabetic populations be included in future studies or that longitudinal studies be planned. Second, this study's sample size is relatively modest. Additionally, the diabetes patients have greater levels of education than the group of NGM, but in the following analysis, the results are still statistically significant after we control this covariable.

The study contains the following strengths. First of all, the analysis of cortical gyrification in patients with T2DM provides information on the relationship between cortical folding patterns and glucose metabolism and cognition. Second, our mediation model enables us to solve the question of whether glucose metabolism markers are independently associated with observed working memory performance deterioration. In order to do this, we employ the bootstrapping approach, which generates a statistical inference test that is not based on the large sample theory and may therefore be utilized even with relatively small sample sizes (Preacher and Hayes 2004).

In conclusion, we found that individuals with T2DM had significant hypogyria in specific brain regions, namely the bilateral temporal pole, left superior temporal cortex, and left transverse temporal cortex compared to NGM. The recent findings may help to better understand how elevated fasting glucose impacts brain anatomy and function, as well as how these two factors interact. Furthermore, our research suggests that enhancing the management of blood glucose levels by lifestyle interventions holds potential as an effective approach to mitigate cognitive loss in older T2DM populations.

## Author Contributions

**Weiye Lu**: Conceptualization, methodology, investigation, software, formal analysis, visualization, writing–original draft. **Yuna Chen**: Investigation, data curation, conceptualization. **Zidong Cao**: Data curation, investigation, software, visualization. **Zhizhong Sun**: Data curation, investigation. **Wenbin Qiu**: Data curation, investigation. **Limin Ge**: Data curation, investigation. **Xin Tan**: Validation, supervision. **Yi Liang**: Writing–review and editing, validation, supervision. **Shijun Qiu**: Validation, supervision, resources, project administration, writing–review and editing, funding acquisition.

## Conflicts of Interest

The authors declare no conflicts of interest.

### Peer Review

The peer review history for this article is available at https://publons.com/publon/10.1002/brb3.70214.

## Supporting information



Appendix E1: Description of the Individual Cognitive Tests Used in the Present Study

Table E1: Multivariable‐adjusted associations of glucose metabolism with statistically different LGIs
Table E2: Multivariable‐adjusted associations of statistically different LGIs with cognitive function
Table E3: Mediation analysis associations of glucose metabolism with cognition, mediated by statistically different LGIs.
Table E4: Comparison of the local gyrification index between type 2 diabetes and normal glucose metabolism after full adjustment

## Data Availability

Some or all datasets generated during and/or analyzed during the current study are not publicly available but are available from the corresponding author upon reasonable request.

## References

[brb370214-bib-0001] American Diabetes Association . 2021. “2. Classification and Diagnosis of Diabetes: Standards of Medical Care in Diabetes—2021.” Diabetes Care 44: Suppl 1: S15–S33. 10.2337/dc21-S002. 33298413

[brb370214-bib-0002] American Diabetes Association . 2015. “(2) Classification and Diagnosis of Diabetes.” Diabetes Care 38: Suppl: S8–S16. 10.2337/dc15-S005. 25537714

[brb370214-bib-0003] Armstrong, E. , A. Schleicher , H. Omran , M. Curtis , and K Zilles . 1995. “The Ontogeny of Human Gyrification.” Cerebral Cortex 5, no. 1: 56–63. 10.1093/cercor/5.1.56. 7719130

[brb370214-bib-0004] Biessels, G. J. , M. W. Strachan , F. L. Visseren , L. J. Kappelle , and R. A. Whitmer . 2014. “Dementia and Cognitive Decline in Type 2 Diabetes and Prediabetic Stages: Towards Targeted Interventions.” Lancet Diabetes & Endocrinology 2, no. 3: 246–255. 10.1016/S2213-8587(13)70088-3. 24622755

[brb370214-bib-0005] Brundel, M. , M. van den Heuvel , J. de Bresser , L. J. Kappelle , G. J. Biessels , and Utrecht Diabetic Encephalopathy Study Group . 2010. “Cerebral Cortical Thickness in Patients With Type 2 Diabetes.” Journal of the Neurological Sciences 299, no. 1–2: 126–130. 10.1016/j.jns.2010.08.048. 20869085

[brb370214-bib-0006] Choi, K. W. , K. M. Han , A. Kim , et al. 2022. “Decreased Cortical Gyrification in Patients With Bipolar Disorder.” Psychological Medicine 52, no. 12: 2232–2244. 10.1017/S0033291720004079. 33190651

[brb370214-bib-0007] Crisóstomo, J. , J. V. Duarte , C. Moreno , L. Gomes , and M. Castelo‐Branco . 2021. “A Novel Morphometric Signature of Brain Alterations in Type 2 Diabetes: Patterns of Changed Cortical Gyrification.” European Journal of Neuroscience 54, no. 6: 6322–6333. 10.1111/ejn.15424. 34390585 PMC9291170

[brb370214-bib-0008] Desikan, R. S. , F. Ségonne , B. Fischl , et al. 2006. “An Automated Labeling System for Subdividing the Human Cerebral Cortex on MRI Scans Into Gyral Based Regions of Interest.” Neuroimage 31, no. 3: 968–980. 10.1016/j.neuroimage.2006.01.021. 16530430

[brb370214-bib-0009] Fischl, B. , and A. M. Dale . 2000. “Measuring the Thickness of the Human Cerebral Cortex From Magnetic Resonance Images.” Proceedings of the National Academy of Sciences of the USA 97, no. 20: 11050–11055. 10.1073/pnas.200033797. 10984517 PMC27146

[brb370214-bib-0010] Garcia, K. E. , C. D. Kroenke , and P. V. Bayly . 2018. “Mechanics of Cortical Folding: Stress, Growth and Stability.” Philosophical Transactions of the Royal Society of London. Series B: Biological Sciences 373, no. 1759: 20170321. 10.1098/rstb.2017.0321. 30249772 PMC6158197

[brb370214-bib-0011] Geijselaers, S. L. C. , S. J. S. Sep , C. D. A. Stehouwer , and G. J. Biessels . 2015. “Glucose Regulation, Cognition, and Brain MRI in Type 2 Diabetes: A Systematic Review.” Lancet Diabetes & Endocrinology 3, no. 1: 75–89. 10.1016/S2213-8587(14)70148-2. 25163604

[brb370214-bib-0012] Güntürkün, O. , F. Ströckens , and S. Ocklenburg . 2020. “Brain Lateralization: A Comparative Perspective.” Physiological Reviews 100, no. 3: 1019–1063. 10.1152/physrev.00006.2019. 32233912

[brb370214-bib-0013] Hayes, A. F. 2013. Introduction to Mediation, Moderation, and Conditional Process Analysis: A Regression‐Based Approach. New York: Guilford.

[brb370214-bib-0014] Herlin, B. , V. Navarro , and S. Dupont . 2021. “The Temporal Pole: From Anatomy to Function‐A Literature Appraisal.” Journal of Chemical Neuroanatomy 113: 101925. 10.1016/j.jchemneu.2021.101925. 33582250

[brb370214-bib-0015] Hsu, J. L. , Y. L. Chen , J. G. Leu , et al. 2012. “Microstructural White Matter Abnormalities in Type 2 Diabetes Mellitus: A Diffusion Tensor Imaging Study.” Neuroimage 59, no. 2: 1098–1105. 10.1016/j.neuroimage.2011.09.041. 21967726

[brb370214-bib-0016] Jing, J. , Y. Zhou , Y. Pan , et al. 2022. “Reduced White Matter Microstructural Integrity in Prediabetes and Diabetes: A Population‐Based Study.” EBioMedicine 82: 104144. 10.1016/j.ebiom.2022.104144. 35810560 PMC9278067

[brb370214-bib-0017] Kerti, L. , A. V. Witte , A. Winkler , U. Grittner , D. Rujescu , and A. Flöel . 2013. “Higher Glucose Levels Associated With Lower Memory and Reduced Hippocampal Microstructure.” Neurology 81, no. 20: 1746–1752. 10.1212/01.wnl.0000435561.00234.ee. 24153444

[brb370214-bib-0018] Lamballais, S. , E. J. Vinke , M. W. Vernooij , M. A. Ikram , and R. L. Muetzel . 2020. “Cortical Gyrification in Relation to Age and Cognition in Older Adults.” Neuroimage 212: 116637. 10.1016/j.neuroimage.2020.116637. 32081782

[brb370214-bib-0019] Luders, E. , P. M. Thompson , K. L. Narr , A. W. Toga , L. Jancke , and C. Gaser . 2006. “A Curvature‐Based Approach to Estimate Local Gyrification on the Cortical Surface.” Neuroimage 29, no. 4: 1224–1230. 10.1016/j.neuroimage.2005.08.049. 16223589

[brb370214-bib-0020] Madan, C. R. 2021. “Age‐Related Decrements in Cortical Gyrification: Evidence From an Accelerated Longitudinal Dataset.” European Journal of Neuroscience 53, no. 5: 1661–1671. 10.1111/ejn.15039. 33171528 PMC7979529

[brb370214-bib-0021] Magliano, D. J. , and E. J Boyko . 2021. IDF Diabetes Atlas 10th Edition Scientific Committee. IDF DIABETES ATLAS. Brussels: International Diabetes Federation. 35914061

[brb370214-bib-0022] Mankovsky, B. , N. Zherdova , E. van den Berg , G. J. Biessels , and J. de Bresser . 2018. “Cognitive Functioning and Structural Brain Abnormalities in People With Type 2 Diabetes Mellitus.” Diabetic Medicine 35, no. 12: 1663–1670. 10.1111/dme.13800. 30230019

[brb370214-bib-0023] Moheet, A. , S. Mangia , and E. R. Seaquist . 2015. “Impact of Diabetes on Cognitive Function and Brain Structure.” Annals of the New York Academy of Sciences 1353: 60–71. 10.1111/nyas.12807. 26132277 PMC4837888

[brb370214-bib-0024] Moran, C. , R. Beare , T. G. Phan , et al. 2015. “Type 2 Diabetes Mellitus and Biomarkers of Neurodegeneration.” Neurology 85, no. 13: 1123–1130. 10.1212/WNL.0000000000001982. 26333802 PMC5573049

[brb370214-bib-0025] Moran, C. , T. G. Phan , J. Chen , et al. 2013. “Brain Atrophy in Type 2 Diabetes: Regional Distribution and Influence on Cognition.” Diabetes Care 36, no. 12: 4036–4042. 10.2337/dc13-0143. 23939539 PMC3836136

[brb370214-bib-0026] Nanda, P. , N. Tandon , I. T. Mathew , et al. 2014. “Local Gyrification Index in Probands With Psychotic Disorders and Their First‐Degree Relatives.” Biological Psychiatry 76, no. 6: 447–455. 10.1016/j.biopsych.2013.11.018. 24369266 PMC4032376

[brb370214-bib-0027] Núñez, C. , A. Callén , F. Lombardini , Y. Compta , and C. Stephan‐Otto . 2020. “Alzheimer's Disease Neuroimaging Initiative. Different Cortical Gyrification Patterns in Alzheimer's Disease and Impact on Memory Performance.” Annals of Neurology 88, no. 1: 67–80. 10.1002/ana.25741. 32277502

[brb370214-bib-0028] Peng, B. , Z. Chen , L. Ma , and Y. Dai . 2015. “Cerebral Alterations of Type 2 Diabetes Mellitus on MRI: A Pilot Study.” Neuroscience Letters 606: 100–105. 10.1016/j.neulet.2015.08.030. 26306652

[brb370214-bib-0029] Preacher, K. J. , and A. F. Hayes . 2004. “SPSS and SAS Procedures for Estimating Indirect Effects in Simple Mediation Models.” Behavior Research Methods, Instruments & Computers 36, no. 4: 717–731. 10.3758/bf03206553. 15641418

[brb370214-bib-0030] Richman, D. P. , R. M. Stewart , J. W. Hutchinson , and V. S. Jr Caviness . 1975. “Mechanical Model of Brain Convolutional Development.” Science 189, no. 4196: 18–21. 10.1126/science.1135626. 1135626

[brb370214-bib-0031] Sadanand, S. , R. Balachandar , and S. Bharath . 2016. “Memory and Executive Functions in Persons with Type 2 Diabetes: A Meta‐Analysis.” Diabetes Metabolism Research and Reviews 32, no. 2: 132–142. 10.1002/dmrr.2664. 25963303

[brb370214-bib-0032] Schaer, M. , M. B. Cuadra , N. Schmansky , B. Fischl , J. P. Thiran , and S. Eliez . 2012. “How to Measure Cortical Folding From MR Images: A Step‐by‐Step Tutorial to Compute Local Gyrification Index.” Journal of Visualized Experiments: JoVE 00, no. 59: e3417. 10.3791/3417. PMC3369773 22230945

[brb370214-bib-0033] Schaer, M. , M. B. Cuadra , L. Tamarit , F. Lazeyras , S. Eliez , and J. P. Thiran . 2008. “A Surface‐Based Approach to Quantify Local Cortical Gyrification.” IEEE Transactions on Medical Imaging 27, no. 2: 161–170. 10.1109/TMI.2007.903576. 18334438

[brb370214-bib-0034] Shao, P. , X. Li , R. Qin , et al. 2022. “Altered Local Gyrification and Functional Connectivity in Type 2 Diabetes Mellitus Patients With Mild Cognitive Impairment: A Pilot Cross‐Sectional Small‐Scale Single Center Study.” Frontiers in Aging Neuroscience 14: 934071. 10.3389/fnagi.2022.934071. 36204559 PMC9530449

[brb370214-bib-0035] van Agtmaal, M. J. M. , A. Houben , V. de Wit , et al. 2018. “Prediabetes Is Associated with Structural Brain Abnormalities: The Maastricht Study.” Diabetes Care 41, no. 12: 2535–2543. 10.2337/dc18-1132. 30327356

[brb370214-bib-0036] van Duinkerken, E. , and C. M. Ryan . 2020. “Diabetes Mellitus in the Young and the Old: Effects on Cognitive Functioning Across the Life Span.” Neurobiology of Disease 134: 104608. 10.1016/j.nbd.2019.104608. 31494283

[brb370214-bib-0037] van Essen, D. C. 1997. “A Tension‐Based Theory of Morphogenesis and Compact Wiring in the Central Nervous System.” Nature 385, no. 6614: 313–318. 10.1038/385313a0. 9002514

[brb370214-bib-0038] van Gennip, A. C. E. , C. D. A. Stehouwer , M. P. J. van Boxtel , et al. 2021. “Association of Type 2 Diabetes, According to the Number of Risk Factors Within Target Range, With Structural Brain Abnormalities, Cognitive Performance, and Risk of Dementia.” Diabetes Care 44, no. 11: 2493–2502. 10.2337/dc21-0149. 34588209 PMC9612883

[brb370214-bib-0039] van Harten, B. , F. E. de Leeuw , H. C. Weinstein , P. Scheltens , and G. J. Biessels . 2006. “Brain Imaging in Patients with Diabetes: A Systematic Review.” Diabetes Care 29, no. 11: 2539–2548. 10.2337/dc06-1637. 17065699

[brb370214-bib-0040] van Sloten, T. T. , S. Sedaghat , M. R. Carnethon , L. J. Launer , and C. D. A. Stehouwer . 2020. “Cerebral Microvascular Complications of Type 2 Diabetes: Stroke, Cognitive Dysfunction, and Depression.” Lancet Diabetes & Endocrinology 8, no. 4: 325–336. 10.1016/S2213-8587(19)30405-X. 32135131 PMC11044807

[brb370214-bib-0041] Vergoossen, L. W. , M. T. Schram , J. J. de Jong , et al. 2020. “White Matter Connectivity Abnormalities in Prediabetes and Type 2 Diabetes: The Maastricht Study.” Diabetes Care 43, no. 1: 201–208. 10.2337/dc19-0762. 31601638

[brb370214-bib-0042] Wennberg, A. M. , A. P. Spira , C. Pettigrew , et al. 2016. “Blood Glucose Levels and Cortical Thinning in Cognitively Normal, Middle‐Aged Adults.” Journal of the Neurological Sciences 365: 89–95. 10.1016/j.jns.2016.04.017. 27206882 PMC4876973

[brb370214-bib-0043] White, T. , and C. C. Hilgetag . 2011. “Gyrification and Neural Connectivity in Schizophrenia.” Development and Psychopathology 23, no. 1: 339–352. 10.1017/S0954579410000842. 21262059

[brb370214-bib-0044] Yao, L. , C. Yang , W. Zhang , et al. 2021. “A Multimodal Meta‐Analysis of Regional Structural and Functional Brain Alterations in Type 2 Diabetes.” Frontiers in Neuroendocrinology 62: 100915. 10.1016/j.yfrne.2021.100915. 33862036

[brb370214-bib-0045] Zhang, J. , Y. Liu , X. Guo , et al. 2022. “Causal Structural Covariance Network Suggesting Structural Alterations Progression in Type 2 Diabetes Patients.” Frontiers in Human Neuroscience 16: 936943. 10.3389/fnhum.2022.936943. 35911591 PMC9336220

[brb370214-bib-0046] Zhang, J. , Y. Wang , J. Wang , et al. 2014. “White Matter Integrity Disruptions Associated with Cognitive Impairments in Type 2 Diabetic Patients.” Diabetes 63, no. 11: 3596–3605. 10.2337/db14-0342. 24947353

[brb370214-bib-0047] Zilles, K. , N. Palomero‐Gallagher , and K. Amunts . 2013. “Development of Cortical Folding During Evolution and Ontogeny.” Trends in Neuroscience (Tins) 36, no. 5: 275–284. 10.1016/j.tins.2013.01.006. 23415112

