# Supplementary material for: Cortical Gyrification and Cognitive Decline in the Human Brain With Type 2 Diabetes Mellitus
Source: Brain Behav. 2025 Jan 20;15(1):e70214. doi: 10.1002/brb3.70214 (PMC11745154; doi:10.1002/brb3.70214)
Supplement: Supplementary file 1 — Appendix E1: Description of the Individual Cognitive Tests Used in the Present Study [file BRB3-15-e70214-s001.docx]

Appendix E1: Description of the Individual Cognitive Tests Used in the Present Study

- 1. **Montreal Cognitive Assessment (MoCA)**

MoCA is a simple and independent cognitive screening tool with excellent sensitivity. It covers multiple cognitive subdomains, including memory function, executive function, language and visual spatial processing, which can be done in 10 minutes and can be placed on one page [50].

- 1. **Auditory Verbal Learning Test**

The test process is as follows: the examiner reads out 12 disyllabic words of three different types, four for each type. Different types of words are randomly presented with an interval of one second. After showing the entire list, participants were asked to recall the words. This learning stage and recall stage are repeated three times. The participants then took a five-minute non-verbal test. Then they were asked to recall the 12 words for the fourth time. The participants were then asked to recall the list for a fifth time after another 20-minute non-verbal task. Finally, participants were asked to identify a word list from 24 words, including 12 real words and 12 interfering words. AVLT short-term recall and long-term recall scored for the fourth and fifth time respectively, and the full score was 12 points respectively [51]. The cognitive domain it assesses is episodic memory.

- 1. **Digital Span Test (backward)**

Subjects were asked to repeat the numbers read by the examiner, starting with two digits, and so on. The more numbers the participants memorized, the greater the number span, and the better short-term memory ability [52]. The cognitive domain it assesses is working memory.

- 1. **Trail Making Test**

The Trail Making Test consists of parts A and B. A: subjects connect the numbered circles with a line in ascending order (from 1 to 25); B: subjects connect the numbers and letters in a cross-over order. Scores are based on the time it takes the subject to complete the task; the longer the time, the worse the cognitive functioning [53]. The cognitive domain it assesses is executive function.

- 1. **Digit Symbol Substitution Test**

Digit Symbol Substitution Test is based on a number-symbol coding table, where each number from 1 to 9 corresponds to a symbol. The subjects matched symbols and numbers according to the coding table as much as possible within 90 seconds. The higher the score, the better the cognitive function [54]. The cognitive domains it assesses include attention and information processing speed.

**REFERENCES**

1. Nasreddine ZS, Phillips NA, Bédirian V, et al. The Montreal Cognitive Assessment, MoCA: a brief screening tool for mild cognitive impairment [published correction appears in J Am Geriatr Soc. 2019 Sep;67(9):1991]. J Am Geriatr Soc. 2005;53(4):695-699. doi:10.1111/j.1532-5415.2005.53221
2. Zhao Q, Guo Q, Liang X, et al. Auditory Verbal Learning Test is Superior to Rey-Osterrieth Complex Figure Memory .for Predicting Mild Cognitive Impairment to Alzheimer's Disease. Curr Alzheimer Res. 2015;12(6):520-526. doi:10.2174/1567205012666150530202729
3. Young JC, Sawyer RJ, Roper BL, Baughman BC. Expansion and re-examination of Digit Span effort indices on the WAIS-IV. Clin Neuropsychol. 2012;26(1):147-159. doi:10.1080/13854046.2011.647083
4. Llinàs-Reglà J, Vilalta-Franch J, López-Pousa S, Calvó-Perxas L, Torrents Rodas D, Garre-Olmo J. The Trail Making Test. Assessment. 2017;24(2):183-196. doi:10.1177/1073191115602552
5. Jaeger J. Digit Symbol Substitution Test: The Case for Sensitivity Over Specificity in Neuropsychological Testing. J .Clin Psychopharmacol. 2018;38(5):513-519. doi:10.1097/JCP.0000000000000941

Table E1: Multivariable-adjusted associations of glucose metabolism with statistically different LGIs

|  | Fasting blood glucose | | HbA1c | | Fasting insulin | |
| --- | --- | --- | --- | --- | --- | --- |
| Statistically different LGIs | Stβ [95%CI] | *P* | Stβ [95%CI] | *P* | Stβ [95%CI] | *P* |
| Left hemisphere |  |  |  |  |  |  |
| Superior temporal cortex | -0.152 (-0.387, 0.083) | 0.201 | -0.162 (-0.395, 0.071) | 0.170 | -0.140 (-0.363, 0.083) | 0.214 |
| Temporal pole | -0.236 (-0.475, 0.003) | 0.053 | -0.021 (-0.264, 0.223) | 0.866 | -0.166 (-0.395, 0.063) | 0.153 |
| Transverse temporal cortex | -0.157 (-0.394, 0.080) | 0.191 | -0.186 (-0.420, 0.048) | 0.118 | -0.093 (-0.319, 0.133) | 0.414 |
| Right hemisphere |  |  |  |  |  |  |
| Temporal pole | **-0.335(-0.547, -0.123)** | **0.002** | -0.017 (-0.240, 0.206) | 0.879 | -0.119 (-0.331, 0.092) | 0.264 |

Data are dispalyed as Standardized β coefficients and 95% CI.

Significant group differences are presented in a bold face.

Table E2: Multivariable-adjusted associations of statistically different LGIs with cognitive function

|  | Left superior temporal cortex | | Left temporal pole | |
| --- | --- | --- | --- | --- |
| Cognition test | Stβ [95%CI] | *P* | Stβ [95%CI] | *P* |
| Moca | -0.010 (-0.202, 0.182) | 0.918 | -0.113 (-0.297, 0.071) | 0.227 |
| Episodic memory | -0.070 (-0.265, 0.126) | 0.480 | -0.076 (-0.265, 0.113) | 0.428 |
| Working memory | 0.026 (-0.184, 0.235) | 0.808 | -0.017 (-0.219, 0.186) | 0.870 |
| Executive function  and attention | -0.189 (-0.398, 0.019) | 0.075 | 0.020 (-0.187, 0.226) | 0.850 |
| Information processing  speed | 0.022 (-0.160, 0.203) | 0.812 | 0.038 (-0.138, 0.214) | 0.667 |
|  | Left transverse temporal cortex | | Right temporal pole | |
| Cognition test | Stβ [95%CI] | *P* | Stβ [95%CI] | *P* |
| Moca | -0.001 (-0.192, 0.189) | 0.988 | -0.060 (-0.262, 0.142) | 0.555 |
| Episodic memory | -0.060 (-0.254, 0.134) | 0.542 | 0.128 (-0.077, 0.333) | 0.217 |
| Working memory | 0.028 (-0.180, 0.235) | 0.790 | **0.268 (0.056, 0.481)** | **0.014** |
| Executive function  and attention | -0.156 (-0.365, 0.052) | 0.139 | 0.050 (-0.174, 0.275) | 0.657 |
| Information processing  speed | 0.005 (-0.175, 0.186) | 0.953 | **0.243 (0.060, 0.427)** | **0.010** |

Data are dispalyed as Standardized β coefficients and 95% CI. Moca, Montreal Cognitive Assessment.

Significant group differences are presented in a bold face.

Table E3: Mediation analysis associations of glucose metabolism with cognition, mediated by statistically different LGIs.

| Y = Moca; M = Right temporal pole LGI | | | | | | | | |
| --- | --- | --- | --- | --- | --- | --- | --- | --- |
|  | Path a | | Path b | | Path c’ | | Indirect effect | PM |
|  | Stβ [95%CI] | *P* | Stβ [95%CI] | *P* | Stβ [95%CI] | *P* | Stβ [95%CI] | [%] |
| FBG | **-0.335(-0.547, -0.123)** | **0.002** | -0.060 (-0.262, 0.142) | 0.555 | **-0.398 (-0.591, -0.206)** | **<0.001** | 0.040 (-0.005, 0.155) | — |
| HbA1c | -0.017 (-0.241, 0.206) | 0.879 | -0.060 (-0.262, 0.142) | 0.555 | **-0.344 (-0.526, -0.162)** | **0.003** | 0.001 (-0.029, 0.026) | — |
| Fasting insulin | -0.120 (-0.331, 0.092) | 0.264 | -0.060 (-0.262, 0.142) | 0.555 | 0.105 (-0.085, 0.295) | 0.275 | 0.006 (-0.037, 0.047) | — |
| Y = Episodic memory; M = Right temporal pole LGI | | | | | | | | |
|  | Path a | | Path b | | Path c’ | | Indirect effect | PM |
|  | Stβ [95%CI] | *P* | Stβ [95%CI] | *P* | Stβ [95%CI] | *P* | Stβ [95%CI] | [%] |
| FBG | **-0.335(-0.547, -0.123)** | **0.002** | 0.128 (-0.077, 0.333) | 0.217 | **-0.239 (-0.448, -0.030)** | **0.025** | -0.015 (-0.099, 0.085) | — |
| HbA1c | -0.017 (-0.241, 0.206) | 0.879 | 0.128 (-0.077, 0.333) | 0.217 | **-0.243 (-0.436, -0.049)** | **0.015** | -0.002 (-0.049, 0.026) | — |
| Fasting insulin | -0.120 (-0.331, 0.092) | 0.264 | 0.128 (-0.077, 0.333) | 0.217 | -0.002 (-0.196, 0.192) | 0.985 | -0.015 (-0.080, 0.037) | — |
| Y = Working memory; M = Right temporal pole LGI | | | | | | | | |
|  | Path a | | Path b | | Path c’ | | Indirect effect | PM |
|  | Stβ [95%CI] | *P* | Stβ [95%CI] | *P* | Stβ [95%CI] | *P* | Stβ [95%CI] | [%] |
| FBG | **-0.335(-0.547, -0.123)** | **0.002** | **0.268 (0.056, 0.481)** | **0.010** | -0.177 (-0.397, 0.043) | 0.114 | **-0.070 (-0.162, -0.002)** | **28.2** |
| HbA1c | -0.017 (-0.241, 0.206) | 0.879 | **0.268 (0.056, 0.481)** | **0.010** | -0.075 (-0.284, 0.133) | 0.473 | -0.005 (-0.080, 0.050) | — |
| Fasting insulin | -0.120 (-0.331, 0.092) | 0.264 | **0.268 (0.056, 0.481)** | **0.010** | -0.051 (-0.252, 0.150) | 0.614 | -0.031 (-0.109, 0.050) | — |
| Y = Executive function and attention; M = Right temporal pole LGI | | | | | | | | |
|  | Path a | | Path b | | Path c’ | | Indirect effect | PM |
|  | Stβ [95%CI] | *P* | Stβ [95%CI] | *P* | Stβ [95%CI] | *P* | Stβ [95%CI] | [%] |
| FBG | **-0.335(-0.547, -0.123)** | **0.002** | 0.050 (-0.174, 0.275) | 0.657 | 0.148 (-0.087, 0.382) | 0.214 | -0.034 (-0.130, 0.061) | — |
| HbA1c | -0.017 (-0.241, 0.206) | 0.879 | 0.050 (-0.174, 0.275) | 0.657 | 0.190 (-0.027, 0.407) | 0.085 | -0.001 (-0.034, 0.023) | — |
| Fasting insulin | -0.120 (-0.331, 0.092) | 0.264 | 0.050 (-0.174, 0.275) | 0.657 | -0.150 (-0.360, 0.061) | 0.160 | -0.004 (-0.046, 0.039) | — |
| Y = Information processing speed; M = Right temporal pole LGI | | | | | | | | |
|  | Path a | | Path b | | Path c’ | | Indirect effect | PM |
|  | Stβ [95%CI] | *P* | Stβ [95%CI] | *P* | Stβ [95%CI] | *P* | Stβ [95%CI] | [%] |
| FBG | **-0.335(-0.547, -0.123)** | **0.002** | **0.243 (0.060, 0.427)** | **0.010** | -0.125 (0.316, 0.067) | 0.198 | -0.067 (-0.159, 0.019) | — |
| HbA1c | -0.017 (-0.241, 0.206) | 0.879 | **0.243 (0.060, 0.427)** | **0.010** | **-0.253 (-0.424, -0.082)** | **0.004** | -0.004 (-0.068, 0.039) | — |
| Fasting insulin | -0.120 (-0.331, 0.092) | 0.264 | **0.243 (0.060, 0.427)** | 0.010 | 0.110 (-0.062, 0.282) | 0.207 | -0.031 (-0.111, 0.056) | — |

Data are dispalyed as Standardized β coefficients and 95% CI. Moca, Montreal Cognitive Assessment.

Significant group differences are presented in a bold face.

| Table E4: Comparison of the local gyrification index between type 2 diabetes and normal glucose metabolism after full adjustment | | | | | | | |
| --- | --- | --- | --- | --- | --- | --- | --- |
|  | T2DM (*n* = 83) | | NGM (*n* = 60) | | T2DM v. NGM | | |
| Cortical regions | Mean | SD | Mean | SD | *F*_(1,137)_ | *P* value | Cohen’s *f*^2^ |
| Left hemisphere |  |  |  |  |  |  |  |
| Caudal anterior cingulate cortex | 1.85 | 0.09 | 1.90 | 0.10 | 1.993 | 0.160 | 0.015 |
| Caudal middle frontal gyrus | 3.06 | 0.16 | 3.09 | 0.15 | 2.294 | 0.132 | 0.017 |
| Cuneus | 2.91 | 0.19 | 2.93 | 0.16 | 0.976 | 0.325 | 0.007 |
| Entorhinal cortex | 2.54 | 0.12 | 2.56 | 0.13 | 0.258 | 0.612 | 0.002 |
| Fusiform gyrus | 2.64 | 0.11 | 2.67 | 0.10 | 0.916 | 0.340 | 0.007 |
| Inferior parietal cortex | 3.19 | 0.13 | 3.24 | 0.12 | 0.039 | 0.943 | 2.9 × 10**^-^**^4^ |
| Inferior temporal gyrus | 2.65 | 0.12 | 2.69 | 0.12 | 1.132 | 0.289 | 0.008 |
| Isthmus of cingulate cortex | 2.68 | 0.17 | 2.74 | 0.18 | 2.665 | 0.105 | 0.020 |
| Lateral occipital cortex | 2.56 | 0.10 | 2.59 | 0.11 | 1.734 | 0.190 | 0.013 |
| Lateral orbitofrontal cortex | 2.53 | 0.10 | 2.58 | 0.11 | 8.807 | 0.004 | 0.062 |
| Lingual gyrus | 2.76 | 0.14 | 2.79 | 0.14 | 0.488 | 0.486 | 0.004 |
| Medial orbitofrontal cortex | 2.07 | 0.09 | 2.12 | 0.09 | 1.768 | 0.186 | 0.013 |
| Middle temporal gyrus | 3.25 | 0.18 | 3.33 | 0.18 | 0.001 | 0.982 | 4.0 × 10**^-^**^6^ |
| Parahippocampal gyrus | 2.82 | 0.14 | 2.82 | 0.15 | 0.522 | 0.471 | 0.004 |
| Paracentral lobule | 2.29 | 0.11 | 2.35 | 0.11 | 1.055 | 0.306 | 0.008 |
| Pars opercularis | 4.02 | 0.28 | 4.10 | 0.26 | 2.388 | 0.125 | 0.018 |
| Pars orbitalis | 2.89 | 0.17 | 2.87 | 0.13 | 0.916 | 0.340 | 0.007 |
| Pars triangularis | 3.60 | 0.23 | 3.65 | 0.25 | 2.314 | 0.131 | 0.017 |
| Pericalcarine cortex | 2.79 | 0.17 | 2.81 | 0.15 | 0.615 | 0.434 | 0.005 |
| Postcentral gyrus | 3.42 | 0.16 | 3.48 | 0.13 | 1.114 | 0.293 | 0.008 |
| Posterior cingulate cortex | 2.14 | 0.13 | 2.18 | 0.14 | 1.590 | 0.210 | 0.012 |
| Precentral gyrus | 3.33 | 0.15 | 3.41 | 0.13 | 3.492 | 0.064 | 0.026 |
| Precuneus | 2.85 | 0.17 | 2.90 | 0.16 | 0.033 | 0.856 | 2.5 × 10**^-^**^4^ |
| Rostral anterior cingulate cortex | 1.99 | 0.08 | 2.05 | 0.09 | 2.896 | 0.091 | 0.021 |
| Rostral middle frontal gyrus | 2.70 | 0.13 | 2.71 | 0.10 | 0.713 | 0.400 | 0.005 |
| Superior frontal gyrus | 2.14 | 0.07 | 2.17 | 0.08 | 1.491 | 0.224 | 0.011 |
| Superior parietal cortex | 2.94 | 0.13 | 2.98 | 0.13 | 0.004 | 0.947 | 3.4 × 10**^-5^** |
| Superior temporal cortex | 3.93 | 0.21 | 4.07 | 0.21 | 3.942 | 0.049 | 0.029 |
| Supramarginal gyrus | 3.50 | 0.15 | 3.56 | 0.14 | 1.074 | 0.302 | 0.008 |
| Frontal pole | 2.04 | 0.09 | 2.07 | 0.09 | 0.650 | 0.422 | 0.005 |
| **Temporal pole** | 2.31 | 0.09 | 2.40 | 0.10 | **14.758** | **1.8× 10^-4^** | 0.100 |
| Transverse temporal cortex | 4.47 | 0.28 | 4.65 | 0.29 | 3.625 | 0.059 | 0.027 |
| Insula | 4.07 | 0.24 | 4.20 | 0.27 | 3.934 | 0.049 | 0.029 |
| Right hemisphere |  |  |  |  |  |  |  |
| Caudal anterior cingulate cortex | 1.92 | 0.10 | 1.94 | 0.11 | 1.967 | 0.163 | 0.015 |
| Caudal middle frontal gyrus | 3.06 | 0.15 | 3.10 | 0.12 | 1.120 | 0.292 | 0.008 |
| Cuneus | 3.07 | 0.19 | 3.12 | 0.20 | 0.156 | 0.693 | 0.001 |
| Entorhinal cortex | 2.58 | 0.12 | 2.61 | 0.13 | 5.208 | 0.024 | 0.038 |
| Fusiform gyrus | 2.63 | 0.11 | 2.64 | 0.11 | 0.159 | 0.690 | 0.001 |
| Inferior parietal cortex | 3.17 | 0.13 | 3.22 | 0.19 | 0.007 | 0.934 | 5.3 × 10**^-5^** |
| Inferior temporal gyrus | 2.59 | 0.10 | 2.61 | 0.08 | 1.347 | 0.248 | 0.010 |
| Isthmus of cingulate cortex | 2.78 | 0.17 | 2.84 | 0.20 | 1.315 | 0.254 | 0.010 |
| Lateral occipital cortex | 2.57 | 0.10 | 2.59 | 0.12 | 3.1 × 10**^-4^** | 0.986 | 2.0 × 10**^-6^** |
| Lateral orbitofrontal cortex | 2.52 | 0.09 | 2.56 | 0.09 | 4.075 | 0.046 | 0.030 |
| Lingual gyrus | 2.84 | 0.14 | 2.89 | 0.15 | 0.769 | 0.382 | 0.006 |
| Medial orbitofrontal cortex | 2.11 | 0.08 | 2.14 | 0.09 | 0.812 | 0.369 | 0.006 |
| Middle temporal gyrus | 3.20 | 0.17 | 3.25 | 0.17 | 0.428 | 0.514 | 0.003 |
| Parahippocampal gyrus | 2.86 | 0.15 | 2.86 | 0.17 | 0.081 | 0.776 | 0.001 |
| Paracentral lobule | 2.30 | 0.10 | 2.37 | 0.11 | 1.631 | 0.204 | 0.012 |
| Pars opercularis | 4.04 | 0.25 | 4.15 | 0.28 | 5.698 | 0.018 | 0.041 |
| Pars orbitalis | 2.91 | 0.17 | 2.92 | 0.16 | 0.211 | 0.647 | 0.002 |
| Pars triangularis | 3.65 | 0.26 | 3.69 | 0.27 | 1.482 | 0.226 | 0.011 |
| Pericalcarine cortex | 2.93 | 0.17 | 2.97 | 0.18 | 0.314 | 0.576 | 0.002 |
| Postcentral gyrus | 3.37 | 0.16 | 3.44 | 0.14 | 0.462 | 0.498 | 0.003 |
| Posterior cingulate cortex | 2.15 | 0.10 | 2.20 | 0.14 | 0.776 | 0.380 | 0.006 |
| Precentral gyrus | 3.31 | 0.15 | 3.38 | 0.12 | 2.072 | 0.152 | 0.015 |
| Precuneus | 2.99 | 0.18 | 3.06 | 0.19 | 1.238 | 0.268 | 0.009 |
| Rostral anterior cingulate cortex | 2.06 | 0.09 | 2.11 | 0.10 | 1.403 | 0.238 | 0.010 |
| Rostral middle frontal gyrus | 2.69 | 0.12 | 2.74 | 0.11 | 1.850 | 0.176 | 0.014 |
| Superior frontal gyrus | 2.21 | 0.08 | 2.25 | 0.07 | 1.316 | 0.253 | 0.010 |
| Superior parietal cortex | 2.92 | 0.12 | 2.98 | 0.12 | 1.223 | 0.271 | 0.009 |
| Superior temporal cortex | 3.95 | 0.22 | 4.05 | 0.24 | 1.096 | 0.297 | 0.008 |
| Supramarginal gyrus | 3.45 | 0.15 | 3.51 | 0.13 | 0.070 | 0.792 | 0.001 |
| Frontal pole | 2.11 | 0.10 | 2.13 | 0.09 | 0.098 | 0.755 | 0.001 |
| **Temporal pole** | 2.31 | 0.10 | 2.38 | 0.10 | **12.046** | **7.0 × 10^-4^** | 0.083 |
| Transverse temporal cortex | 4.49 | 0.27 | 4.66 | 0.30 | 4.571 | 0.034 | 0.033 |
| Insula | 4.08 | 0.25 | 4.18 | 0.28 | 2.606 | 0.109 | 0.019 |
| Mean LGI of the whole brain | 2.91 | 0.10 | 2.96 | 0.09 | 1.949 | 0.165 | 0.014 |

T2DM, type 2 diabetes mellitus, NGM, normal glucose metabolism; SD, standard deviation.

Local gyrification index (LGI) are displayed as mean with one standard deviation.

Significant between-groups differences after Bonferroni correction (*P* < 0.05/66 = 0.000758) are shown in bold.
