# Supplementary material for: Cortical Gyrification and Cognitive Decline in the Human Brain With Type 2 Diabetes Mellitus
Source: Brain Behav. 2025 Jan 20;15(1):e70214. doi: 10.1002/brb3.70214 (PMC11745154; doi:10.1002/brb3.70214)
Supplement: Supplementary file 2 — Table E1: Multivariable‐adjusted associations of glucose metabolism with statistically different LGIs Table E2: Multivariable‐adjusted associations of statistically different LGIs with cognitive function Table E3: Mediation analysis associations of glucose metabolism with cognition, mediated by statistically different LGIs. Table E4: Comparison of the local gyrification index between type 2 diabetes and normal glucose metabolism after full adjustment [file BRB3-15-e70214-s002.docx]

Table E1: Multivariable-adjusted associations of glucose metabolism with statistically different LGIs

|  | Fasting blood glucose | | HbA1c | | Fasting insulin | |
| --- | --- | --- | --- | --- | --- | --- |
| Statistically different LGIs | Stβ [95%CI] | *P* | Stβ [95%CI] | *P* | Stβ [95%CI] | *P* |
| Left hemisphere |  |  |  |  |  |  |
| Superior temporal cortex | -0.152 (-0.387, 0.083) | 0.201 | -0.162 (-0.395, 0.071) | 0.170 | -0.140 (-0.363, 0.083) | 0.214 |
| Temporal pole | -0.236 (-0.475, 0.003) | 0.053 | -0.021 (-0.264, 0.223) | 0.866 | -0.166 (-0.395, 0.063) | 0.153 |
| Transverse temporal cortex | -0.157 (-0.394, 0.080) | 0.191 | -0.186 (-0.420, 0.048) | 0.118 | -0.093 (-0.319, 0.133) | 0.414 |
| Right hemisphere |  |  |  |  |  |  |
| Temporal pole | **-0.335(-0.547, -0.123)** | **0.002** | -0.017 (-0.240, 0.206) | 0.879 | -0.119 (-0.331, 0.092) | 0.264 |

Data are dispalyed as Standardized β coefficients and 95% CI.

Significant group differences are presented in a bold face.

Table E2: Multivariable-adjusted associations of statistically different LGIs with cognitive function

|  | Left superior temporal cortex | | Left temporal pole | |
| --- | --- | --- | --- | --- |
| Cognition test | Stβ [95%CI] | *P* | Stβ [95%CI] | *P* |
| Moca | -0.010 (-0.202, 0.182) | 0.918 | -0.113 (-0.297, 0.071) | 0.227 |
| Episodic memory | -0.070 (-0.265, 0.126) | 0.480 | -0.076 (-0.265, 0.113) | 0.428 |
| Working memory | 0.026 (-0.184, 0.235) | 0.808 | -0.017 (-0.219, 0.186) | 0.870 |
| Executive function  and attention | -0.189 (-0.398, 0.019) | 0.075 | 0.020 (-0.187, 0.226) | 0.850 |
| Information processing  speed | 0.022 (-0.160, 0.203) | 0.812 | 0.038 (-0.138, 0.214) | 0.667 |
|  | Left transverse temporal cortex | | Right temporal pole | |
| Cognition test | Stβ [95%CI] | *P* | Stβ [95%CI] | *P* |
| Moca | -0.001 (-0.192, 0.189) | 0.988 | -0.060 (-0.262, 0.142) | 0.555 |
| Episodic memory | -0.060 (-0.254, 0.134) | 0.542 | 0.128 (-0.077, 0.333) | 0.217 |
| Working memory | 0.028 (-0.180, 0.235) | 0.790 | **0.268 (0.056, 0.481)** | **0.014** |
| Executive function  and attention | -0.156 (-0.365, 0.052) | 0.139 | 0.050 (-0.174, 0.275) | 0.657 |
| Information processing  speed | 0.005 (-0.175, 0.186) | 0.953 | **0.243 (0.060, 0.427)** | **0.010** |

Data are dispalyed as Standardized β coefficients and 95% CI. Moca, Montreal Cognitive Assessment.

Significant group differences are presented in a bold face.

Table E3: Mediation analysis associations of glucose metabolism with cognition, mediated by statistically different LGIs.

| Y = Moca; M = Right temporal pole LGI | | | | | | | | |
| --- | --- | --- | --- | --- | --- | --- | --- | --- |
|  | Path a | | Path b | | Path c’ | | Indirect effect | PM |
|  | Stβ [95%CI] | *P* | Stβ [95%CI] | *P* | Stβ [95%CI] | *P* | Stβ [95%CI] | [%] |
| FBG | **-0.335(-0.547, -0.123)** | **0.002** | -0.060 (-0.262, 0.142) | 0.555 | **-0.398 (-0.591, -0.206)** | **<0.001** | 0.040 (-0.005, 0.155) | — |
| HbA1c | -0.017 (-0.241, 0.206) | 0.879 | -0.060 (-0.262, 0.142) | 0.555 | **-0.344 (-0.526, -0.162)** | **0.003** | 0.001 (-0.029, 0.026) | — |
| Fasting insulin | -0.120 (-0.331, 0.092) | 0.264 | -0.060 (-0.262, 0.142) | 0.555 | 0.105 (-0.085, 0.295) | 0.275 | 0.006 (-0.037, 0.047) | — |
| Y = Episodic memory; M = Right temporal pole LGI | | | | | | | | |
|  | Path a | | Path b | | Path c’ | | Indirect effect | PM |
|  | Stβ [95%CI] | *P* | Stβ [95%CI] | *P* | Stβ [95%CI] | *P* | Stβ [95%CI] | [%] |
| FBG | **-0.335(-0.547, -0.123)** | **0.002** | 0.128 (-0.077, 0.333) | 0.217 | **-0.239 (-0.448, -0.030)** | **0.025** | -0.015 (-0.099, 0.085) | — |
| HbA1c | -0.017 (-0.241, 0.206) | 0.879 | 0.128 (-0.077, 0.333) | 0.217 | **-0.243 (-0.436, -0.049)** | **0.015** | -0.002 (-0.049, 0.026) | — |
| Fasting insulin | -0.120 (-0.331, 0.092) | 0.264 | 0.128 (-0.077, 0.333) | 0.217 | -0.002 (-0.196, 0.192) | 0.985 | -0.015 (-0.080, 0.037) | — |
| Y = Working memory; M = Right temporal pole LGI | | | | | | | | |
|  | Path a | | Path b | | Path c’ | | Indirect effect | PM |
|  | Stβ [95%CI] | *P* | Stβ [95%CI] | *P* | Stβ [95%CI] | *P* | Stβ [95%CI] | [%] |
| FBG | **-0.335(-0.547, -0.123)** | **0.002** | **0.268 (0.056, 0.481)** | **0.010** | -0.177 (-0.397, 0.043) | 0.114 | **-0.070 (-0.162, -0.002)** | **28.2** |
| HbA1c | -0.017 (-0.241, 0.206) | 0.879 | **0.268 (0.056, 0.481)** | **0.010** | -0.075 (-0.284, 0.133) | 0.473 | -0.005 (-0.080, 0.050) | — |
| Fasting insulin | -0.120 (-0.331, 0.092) | 0.264 | **0.268 (0.056, 0.481)** | **0.010** | -0.051 (-0.252, 0.150) | 0.614 | -0.031 (-0.109, 0.050) | — |
| Y = Executive function and attention; M = Right temporal pole LGI | | | | | | | | |
|  | Path a | | Path b | | Path c’ | | Indirect effect | PM |
|  | Stβ [95%CI] | *P* | Stβ [95%CI] | *P* | Stβ [95%CI] | *P* | Stβ [95%CI] | [%] |
| FBG | **-0.335(-0.547, -0.123)** | **0.002** | 0.050 (-0.174, 0.275) | 0.657 | 0.148 (-0.087, 0.382) | 0.214 | -0.034 (-0.130, 0.061) | — |
| HbA1c | -0.017 (-0.241, 0.206) | 0.879 | 0.050 (-0.174, 0.275) | 0.657 | 0.190 (-0.027, 0.407) | 0.085 | -0.001 (-0.034, 0.023) | — |
| Fasting insulin | -0.120 (-0.331, 0.092) | 0.264 | 0.050 (-0.174, 0.275) | 0.657 | -0.150 (-0.360, 0.061) | 0.160 | -0.004 (-0.046, 0.039) | — |
| Y = Information processing speed; M = Right temporal pole LGI | | | | | | | | |
|  | Path a | | Path b | | Path c’ | | Indirect effect | PM |
|  | Stβ [95%CI] | *P* | Stβ [95%CI] | *P* | Stβ [95%CI] | *P* | Stβ [95%CI] | [%] |
| FBG | **-0.335(-0.547, -0.123)** | **0.002** | **0.243 (0.060, 0.427)** | **0.010** | -0.125 (0.316, 0.067) | 0.198 | -0.067 (-0.159, 0.019) | — |
| HbA1c | -0.017 (-0.241, 0.206) | 0.879 | **0.243 (0.060, 0.427)** | **0.010** | **-0.253 (-0.424, -0.082)** | **0.004** | -0.004 (-0.068, 0.039) | — |
| Fasting insulin | -0.120 (-0.331, 0.092) | 0.264 | **0.243 (0.060, 0.427)** | 0.010 | 0.110 (-0.062, 0.282) | 0.207 | -0.031 (-0.111, 0.056) | — |

Data are dispalyed as Standardized β coefficients and 95% CI. Moca, Montreal Cognitive Assessment.

Significant group differences are presented in a bold face.

| Table E4: Comparison of the local gyrification index between type 2 diabetes and normal glucose metabolism after full adjustment | | | | | | | |
| --- | --- | --- | --- | --- | --- | --- | --- |
|  | T2DM (*n* = 83) | | NGM (*n* = 60) | | T2DM v. NGM | | |
| Cortical regions | Mean | SD | Mean | SD | *F*_(1,137)_ | *P* value | Cohen’s *f*^2^ |
| Left hemisphere |  |  |  |  |  |  |  |
| Caudal anterior cingulate cortex | 1.85 | 0.09 | 1.90 | 0.10 | 1.993 | 0.160 | 0.015 |
| Caudal middle frontal gyrus | 3.06 | 0.16 | 3.09 | 0.15 | 2.294 | 0.132 | 0.017 |
| Cuneus | 2.91 | 0.19 | 2.93 | 0.16 | 0.976 | 0.325 | 0.007 |
| Entorhinal cortex | 2.54 | 0.12 | 2.56 | 0.13 | 0.258 | 0.612 | 0.002 |
| Fusiform gyrus | 2.64 | 0.11 | 2.67 | 0.10 | 0.916 | 0.340 | 0.007 |
| Inferior parietal cortex | 3.19 | 0.13 | 3.24 | 0.12 | 0.039 | 0.943 | 2.9 × 10**^-^**^4^ |
| Inferior temporal gyrus | 2.65 | 0.12 | 2.69 | 0.12 | 1.132 | 0.289 | 0.008 |
| Isthmus of cingulate cortex | 2.68 | 0.17 | 2.74 | 0.18 | 2.665 | 0.105 | 0.020 |
| Lateral occipital cortex | 2.56 | 0.10 | 2.59 | 0.11 | 1.734 | 0.190 | 0.013 |
| Lateral orbitofrontal cortex | 2.53 | 0.10 | 2.58 | 0.11 | 8.807 | 0.004 | 0.062 |
| Lingual gyrus | 2.76 | 0.14 | 2.79 | 0.14 | 0.488 | 0.486 | 0.004 |
| Medial orbitofrontal cortex | 2.07 | 0.09 | 2.12 | 0.09 | 1.768 | 0.186 | 0.013 |
| Middle temporal gyrus | 3.25 | 0.18 | 3.33 | 0.18 | 0.001 | 0.982 | 4.0 × 10**^-^**^6^ |
| Parahippocampal gyrus | 2.82 | 0.14 | 2.82 | 0.15 | 0.522 | 0.471 | 0.004 |
| Paracentral lobule | 2.29 | 0.11 | 2.35 | 0.11 | 1.055 | 0.306 | 0.008 |
| Pars opercularis | 4.02 | 0.28 | 4.10 | 0.26 | 2.388 | 0.125 | 0.018 |
| Pars orbitalis | 2.89 | 0.17 | 2.87 | 0.13 | 0.916 | 0.340 | 0.007 |
| Pars triangularis | 3.60 | 0.23 | 3.65 | 0.25 | 2.314 | 0.131 | 0.017 |
| Pericalcarine cortex | 2.79 | 0.17 | 2.81 | 0.15 | 0.615 | 0.434 | 0.005 |
| Postcentral gyrus | 3.42 | 0.16 | 3.48 | 0.13 | 1.114 | 0.293 | 0.008 |
| Posterior cingulate cortex | 2.14 | 0.13 | 2.18 | 0.14 | 1.590 | 0.210 | 0.012 |
| Precentral gyrus | 3.33 | 0.15 | 3.41 | 0.13 | 3.492 | 0.064 | 0.026 |
| Precuneus | 2.85 | 0.17 | 2.90 | 0.16 | 0.033 | 0.856 | 2.5 × 10**^-^**^4^ |
| Rostral anterior cingulate cortex | 1.99 | 0.08 | 2.05 | 0.09 | 2.896 | 0.091 | 0.021 |
| Rostral middle frontal gyrus | 2.70 | 0.13 | 2.71 | 0.10 | 0.713 | 0.400 | 0.005 |
| Superior frontal gyrus | 2.14 | 0.07 | 2.17 | 0.08 | 1.491 | 0.224 | 0.011 |
| Superior parietal cortex | 2.94 | 0.13 | 2.98 | 0.13 | 0.004 | 0.947 | 3.4 × 10**^-5^** |
| Superior temporal cortex | 3.93 | 0.21 | 4.07 | 0.21 | 3.942 | 0.049 | 0.029 |
| Supramarginal gyrus | 3.50 | 0.15 | 3.56 | 0.14 | 1.074 | 0.302 | 0.008 |
| Frontal pole | 2.04 | 0.09 | 2.07 | 0.09 | 0.650 | 0.422 | 0.005 |
| **Temporal pole** | 2.31 | 0.09 | 2.40 | 0.10 | **14.758** | **1.8× 10^-4^** | 0.100 |
| Transverse temporal cortex | 4.47 | 0.28 | 4.65 | 0.29 | 3.625 | 0.059 | 0.027 |
| Insula | 4.07 | 0.24 | 4.20 | 0.27 | 3.934 | 0.049 | 0.029 |
| Right hemisphere |  |  |  |  |  |  |  |
| Caudal anterior cingulate cortex | 1.92 | 0.10 | 1.94 | 0.11 | 1.967 | 0.163 | 0.015 |
| Caudal middle frontal gyrus | 3.06 | 0.15 | 3.10 | 0.12 | 1.120 | 0.292 | 0.008 |
| Cuneus | 3.07 | 0.19 | 3.12 | 0.20 | 0.156 | 0.693 | 0.001 |
| Entorhinal cortex | 2.58 | 0.12 | 2.61 | 0.13 | 5.208 | 0.024 | 0.038 |
| Fusiform gyrus | 2.63 | 0.11 | 2.64 | 0.11 | 0.159 | 0.690 | 0.001 |
| Inferior parietal cortex | 3.17 | 0.13 | 3.22 | 0.19 | 0.007 | 0.934 | 5.3 × 10**^-5^** |
| Inferior temporal gyrus | 2.59 | 0.10 | 2.61 | 0.08 | 1.347 | 0.248 | 0.010 |
| Isthmus of cingulate cortex | 2.78 | 0.17 | 2.84 | 0.20 | 1.315 | 0.254 | 0.010 |
| Lateral occipital cortex | 2.57 | 0.10 | 2.59 | 0.12 | 3.1 × 10**^-4^** | 0.986 | 2.0 × 10**^-6^** |
| Lateral orbitofrontal cortex | 2.52 | 0.09 | 2.56 | 0.09 | 4.075 | 0.046 | 0.030 |
| Lingual gyrus | 2.84 | 0.14 | 2.89 | 0.15 | 0.769 | 0.382 | 0.006 |
| Medial orbitofrontal cortex | 2.11 | 0.08 | 2.14 | 0.09 | 0.812 | 0.369 | 0.006 |
| Middle temporal gyrus | 3.20 | 0.17 | 3.25 | 0.17 | 0.428 | 0.514 | 0.003 |
| Parahippocampal gyrus | 2.86 | 0.15 | 2.86 | 0.17 | 0.081 | 0.776 | 0.001 |
| Paracentral lobule | 2.30 | 0.10 | 2.37 | 0.11 | 1.631 | 0.204 | 0.012 |
| Pars opercularis | 4.04 | 0.25 | 4.15 | 0.28 | 5.698 | 0.018 | 0.041 |
| Pars orbitalis | 2.91 | 0.17 | 2.92 | 0.16 | 0.211 | 0.647 | 0.002 |
| Pars triangularis | 3.65 | 0.26 | 3.69 | 0.27 | 1.482 | 0.226 | 0.011 |
| Pericalcarine cortex | 2.93 | 0.17 | 2.97 | 0.18 | 0.314 | 0.576 | 0.002 |
| Postcentral gyrus | 3.37 | 0.16 | 3.44 | 0.14 | 0.462 | 0.498 | 0.003 |
| Posterior cingulate cortex | 2.15 | 0.10 | 2.20 | 0.14 | 0.776 | 0.380 | 0.006 |
| Precentral gyrus | 3.31 | 0.15 | 3.38 | 0.12 | 2.072 | 0.152 | 0.015 |
| Precuneus | 2.99 | 0.18 | 3.06 | 0.19 | 1.238 | 0.268 | 0.009 |
| Rostral anterior cingulate cortex | 2.06 | 0.09 | 2.11 | 0.10 | 1.403 | 0.238 | 0.010 |
| Rostral middle frontal gyrus | 2.69 | 0.12 | 2.74 | 0.11 | 1.850 | 0.176 | 0.014 |
| Superior frontal gyrus | 2.21 | 0.08 | 2.25 | 0.07 | 1.316 | 0.253 | 0.010 |
| Superior parietal cortex | 2.92 | 0.12 | 2.98 | 0.12 | 1.223 | 0.271 | 0.009 |
| Superior temporal cortex | 3.95 | 0.22 | 4.05 | 0.24 | 1.096 | 0.297 | 0.008 |
| Supramarginal gyrus | 3.45 | 0.15 | 3.51 | 0.13 | 0.070 | 0.792 | 0.001 |
| Frontal pole | 2.11 | 0.10 | 2.13 | 0.09 | 0.098 | 0.755 | 0.001 |
| **Temporal pole** | 2.31 | 0.10 | 2.38 | 0.10 | **12.046** | **7.0 × 10^-4^** | 0.083 |
| Transverse temporal cortex | 4.49 | 0.27 | 4.66 | 0.30 | 4.571 | 0.034 | 0.033 |
| Insula | 4.08 | 0.25 | 4.18 | 0.28 | 2.606 | 0.109 | 0.019 |
| Mean LGI of the whole brain | 2.91 | 0.10 | 2.96 | 0.09 | 1.949 | 0.165 | 0.014 |

T2DM, type 2 diabetes mellitus, NGM, normal glucose metabolism; SD, standard deviation.

Local gyrification index (LGI) are displayed as mean with one standard deviation.

Significant between-groups differences after Bonferroni correction (*P* < 0.05/66 = 0.000758) are shown in bold.
